# Supplementary material for: Bromo- and iodo-bridged building units in metal-organic frameworks for enhanced carrier transport and CO2 photoreduction by water vapor
Source: Nat Commun. 2022 Aug 6;13:4592. doi: 10.1038/s41467-022-32367-0 (PMC9357079; doi:10.1038/s41467-022-32367-0)
Supplement: Supplementary file 1 — Supplementary Information [file 41467_2022_32367_MOESM1_ESM.pdf]

# Supplementary Information

# Bromo- and Iodo-Bridged Building Units in Metal-Organic Frameworks for Enhanced Carrier Transport and $CO_2$ Photoreduction by Water Vapor

*Xinfeng Chen,*[1] *Chendong Peng,*[1] *Wenyan Dan,*[1] *Long Yu,*[1] *Yinan Wu,*[2,3] *and Honghan Fei*[1,*]

[1]School of Chemical Science and Engineering, Shanghai Key Laboratory of Chemical Assessment and Sustainability, Tongji University, Shanghai 200092, P. R. China

[2]College of Environmental Science and Engineering, State Key Laboratory of Pollution Control and Resource Reuse, Tongji University, Shanghai 200092, P. R. China

[3]Shanghai Institute of Pollution Control and Ecological Security, Shanghai 200092, P. R. China

* Corresponding author: fei@tongji.edu.cn

## Supplementary Tables and Figures

**Supplementary Table 1** Crystal data and structure refinement for TMOF-10-$NH_2$(I) and TMOF-10-$NH_2$(Br).

| Identification code | TMOF-10-$NH_2$(I) | TMOF-10-$NH_2$(Br) |
|---|---|---|
| Empirical formula | $C_{18}H_{18}IN_3O_8Pb_2$ | $C_{18}H_{18}BrN_3O_8Pb_2$ |
| Formula weight | 945.63 | 898.645 |
| Temperature/K | 296(2) | 296(2) |
| Crystal system | monoclinic | monoclinic |
| Space group | $P2_1/c$ | $P2_1/c$ |
| Unit cell dimensions | $a$ = 8.0582(10) Å<br>$b$ = 20.334(3) Å<br>$c$ = 28.703(4) Å<br>$\alpha$ = 90°<br>$\beta$ = 95.003(4)°<br>$\gamma$ = 90° | $a$ = 8.0112(5) Å<br>$b$ = 20.3569(11) Å<br>$c$ = 27.7087(16) Å<br>$\alpha$ = 90°<br>$\beta$ = 95.592(2)°<br>$\gamma$ = 90° |
| Volume/Å$^3$, $Z$ | 4685.3(11), 4 | 4497.3(5), 4 |
| Density (calculated) (g cm$^{-3}$) | 2.681 | 2.654 |
| μ/mm$^{-1}$ | 15.720 | 16.781 |
| $F$ (000) | 3424.0 | 3280.0 |
| Crystal size | 0.34 × 0.12 × 0.08 | 0.19 × 0.08 × 0.02 |
| Radiation | MoKα (λ = 0.71073) | MoKα (λ = 0.71073) |
| 2θ range /deg | 5.858 to 49.998 | 5.11 to 53.498 |
| Limiting indices | $-8 \le h \le 9$,<br>$-24 \le k \le 24$,<br>$-34 \le l \le 34$ | $-10 \le h \le 10$,<br>$-25 \le k \le 25$,<br>$-35 \le l \le 35$ |
| Reflections collected | 55289 | 57344 |
| Independent reflections | 8250 [$R$int = 0.0497] | 9542 [$R$int = 0.0837] |
| Data / restraints / parameters | 8250/1941/654 | 9542/1663/626 |
| Goodness-of-fit on $F^2$ | 1.216 | 1.012 |
| Final R indices [I > 2$\sigma$ (I)] | $R_1$ = 0.0525, $wR_2$ = 0.1010 | $R_1$ = 0.0415, $wR_2$ = 0.0764 |
| Final R indices (all data) | $R_1$ = 0.0751, $wR_2$ = 0.1084 | $R_1$ =0.0924, $wR_2$ = 0.0944 |
| Largest diff. peak/hole /e Å$^{-3}$ | 1.55/-1.71 | 1.50/-1.22 |

$R_1=\sum(||F_o|-|F_c||)/\sum|F_o|$; $wR_2=\{\sum[w(F_o^2-F_c^2)]/\sum[w(F_o^2)]^2\}^{1/2}$

**Supplementary Table 2** Crystal data and structure refinement for $[Pb(NH_2\text{-bdc})]_n$.

| Identification code | $[Pb(NH_2\text{-bdc})]_n$ |
|---|---|
| Empirical formula | $C_8H_5NO_4Pb$ |
| Formula weight | 386.32 |
| Temperature/K | 296(2) |
| Crystal system | monoclinic |
| Space group | $P2_1/c$ |
| Unit cell dimensions | $a$ = 20.643(4) Å<br>$b$ = 17.356(4) Å<br>$c$ = 15.380(3) Å<br>$\alpha$ = 90°<br>$\beta$ = 90.028(6)°<br>$\gamma$ = 90° |
| Volume/Å$^3$, $Z$ | 5510.4(19), 19 |
| Density (calculated) (g cm$^{-3}$) | 1.863 |
| μ/mm$^{-1}$ | 12.237 |
| $F$ (000) | 2784.0 |
| Crystal size | 0.35 × 0.21 × 0.13 |
| Radiation | MoKα (λ = 0.71073) |
| 2θ range /deg | 3.538 to 50.05 |
| Limiting indices | $-24 \leq h \leq 20$,<br>$-19 \leq k \leq 20$,<br>$-18 \leq l \leq 18$ |
| Reflections collected | 35436 |
| Independent reflections | 9731 [$R$int = 0.0856] |
| Data / restraints / parameters | 9731/458/485 |
| Goodness-of-fit on $F^2$ | 1.040 |
| Final R indices [I > 2$\sigma$ (I)] | $R_1$ = 0.0673, $wR_2$ = 0.1765 |
| Final R indices (all data) | $R_1$ = 0.0809, $wR_2$ = 0.1857 |
| Largest diff. peak/hole /e Å$^{-3}$ | 3.02/-3.05 |

$R_1=\sum(||F_o|-|F_c||) / \sum|F_o|$; $wR_2=\{\sum[w(F_o^2-F_c^2)]/\sum[w(F_o^2)]^2\}^{1/2}$

**Supplementary Table 3** A summary of photocatalytic $CO_2$ reduction performances by the reported MOF-based catalysts using $H_2O$ as the electron donor.

| Catalyst | Light source | Reaction medium | Rate (μmol $h^{-1}g^{-1}$) | Ref. |
|---|---|---|---|---|
| Co-ZIF-9/$TiO_2$ | 200 nm ≤ λ≤ 900 nm | $H_2O$ | 17.6 (CO)<br>2.0 ($CH_4$) | 1 |
| (Zr-PMOF)/ultrathin g-$C_3N_4$ | λ > 420 nm | $H_2O$ | 5.5 (CO) | 2 |
| $Zn_2GeO_4$/ZIF-8 | 500 W Xe-lamp | $H_2O$ | 0.22 ($CH_3OH$) | 3 |
| $TiO_2$/UiO-66($NH_2$) | 150 W Xe-lamp | $H_2O$ vapor | 8.4 (CO) | 4 |
| CPO-27 Mg/$TiO_2$ | 16 W laser<br>365 nm | $H_2O$ vapor | 4.09 (CO)<br>2.35 ($CH_4$) | 5 |
| $TiO_2$-$Cu_3(BTC)_2$ | UV light λ < 400<br>300 W Xe-lamp | $H_2O$ vapor | 2.64 ($CH_4$) | 6 |
| PCN-601 | 300W Xe-lamp | $H_2O$ vapor | 92.0 ($CH_4$) | 7 |
| NNU-31-Zn | 420 nm ≤ λ≤ 800 nm | $H_2O$ | 26.3 (HCOOH) | 8 |
| FeTCP-OH-Co | λ > 420 nm | $H_2O$ | 17.72 (HCOOH) | 9 |
| **Ru@TMOF-10-$NH_2$(I)** | **300 W Xe-lamp** | **$H_2O$ vapor** | **154 (CO)** | **This work** |

**Supplementary Table 4** A summary of photocatalytic $CO_2$ reduction performances by the reported lead halide hybrids using $H_2O$ as the electron donor.

| Catalyst | Light source | Reaction medium | Rate (μmol $h^{-1}g^{-1}$) | Ref. |
|---|---|---|---|---|
| Fe(II)-doped $CsPbBr_3$ | 450 W Xe-lamp 150 mW $cm^{-2}$ | $H_2O$ vapor | 3.2 (CO) 6.1 ($CH_4$) | 10 |
| Pb-rich $Ni^{2+}$-doped $CsPbCl_3$QDs | 150 mW $cm^{-2}$ AM 1.5G filter | $H_2O$ vapor | 169.37 (CO) | 11 |
| $CsPbBr_3$/ $Cs_4PbBr_6$@Co | 100 mW $cm^{-2}$ $\lambda > 420$ nm | $H_2O$ | 11.95 (CO) | 12 |
| $CsPbBr_3$ NC /ZnO NW/rGO | 150 mW $cm^{-2}$ AM 1.5G filter | $H_2O$ vapor | 6.29 (CO) | 13 |
| $CsPbBr_3$/α- $Fe_2O_3$ rods/amine-rGO | 150 mW $cm^{-2}$ $\lambda > 420$ nm | $H_2O$ vapor | 2.36 (CO) 9.45 ($CH_4$) | 14 |
| $CsPbBr_3$ NCs/Pd NSs | 150 mW $cm^{-2}$ $\lambda > 420$ nm | $H_2O$ vapor | 1.92 (CO) 3.47 ($CH_4$) | 15 |
| $CsPbBr_3$/ZIF | 150 mW $cm^{-2}$ AM 1.5G filter | $H_2O$ vapor | 4.09 (CO) 2.35 ($CH_4$) | 16 |
| $MAPbI_3$@PCN-221(Fe) | 300W Xe-lamp | $H_2O$ | 6.6 (CO) 12.9 ($CH_4$) | 17 |
| **Ru@TMOF-10-$NH_2$(I)** | **300 W Xe-lamp** | **$H_2O$ vapor** | **154 (CO)** | **This work** |

**Supplementary Table 5** Elemental analysis and ICP results of TMOF-10-$NH_2$(I).

| | Pb | C | N | H | O |
|---|---|---|---|---|---|
| **TMOF-10-$NH_2$(I) (calculated)** | **8.84%** | **22.86%** | **4.44%** | **1.90%** | **13.54%** |
| **TMOF-10-$NH_2$(I) (observed)** | **8.81% (by ICP)** | **22.96% (by EA)** | **4.47% (by EA)** | **1.91% (by EA)** | **13.47% (by EA)** |

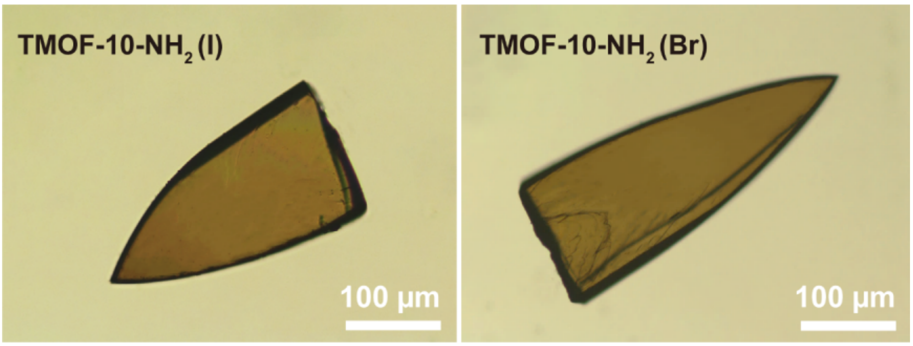


**Supplementary Figure 1 | Optical microscope images of TMOF-10-$NH_2$.** Optical microscope images of TMOF-10-$NH_2$(I) and TMOF-10-$NH_2$(Br).

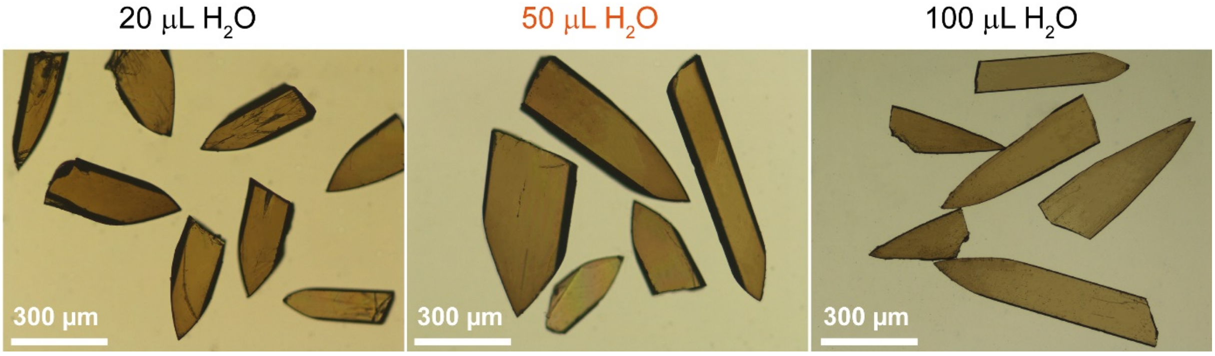


**Supplementary Figure 2 | Optical microscope images of TMOF-10-$NH_2$(I).** Optical microscope images of TMOF-10-$NH_2$(I) crystals synthesized in the presence of different amounts of $H_2O$.

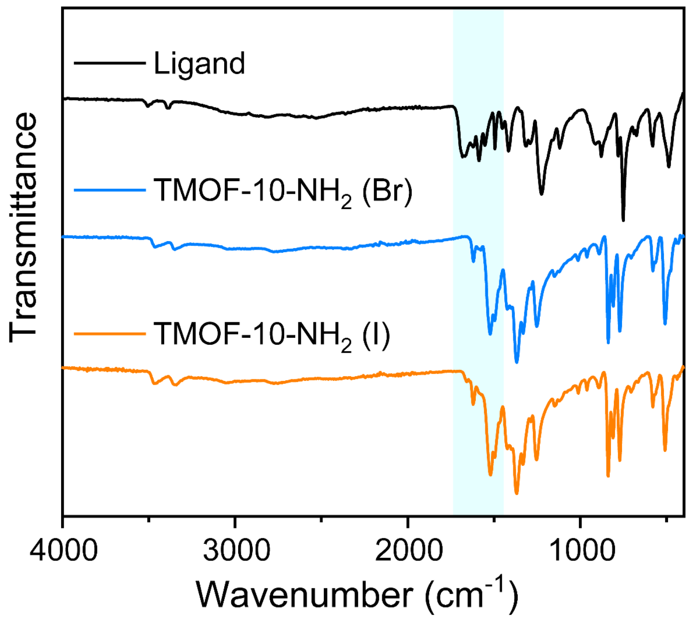


**Supplementary Figure 3 | FT-IR spectra of $NH_2$-bdc and TMOF-10-$NH_2$.** FT-IR spectra of $NH_2$-bdc (black), as-synthesized TMOF-10-$NH_2$(Br) (blue) and TMOF-10-$NH_2$(I) (orange).

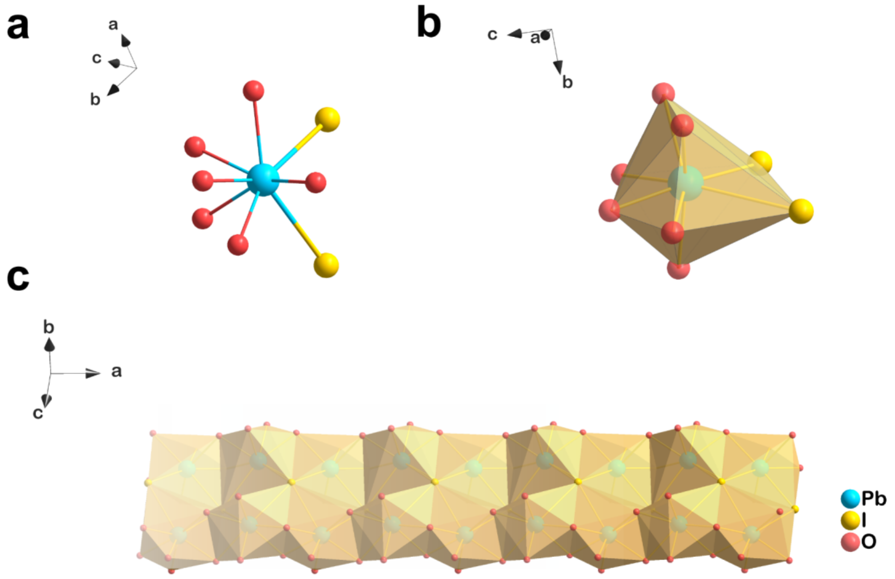


**Supplementary Figure 4 | Crystallographic view of TMOF-10-$NH_2$(I) a,** $PbI_2O_6$ coordination units in TMOF-10-$NH_2$(I). **b,** Crystallgraphic view of a single $Pb^{2+}$-centered dodecahedron in TMOF-10-$NH_2$(I). **c,** Crystallographic view of a lead oxyiodide chain with plane-sharing $Pb^{2+}$-centered dodecahedrons in TMOF-10-$NH_2$(I).

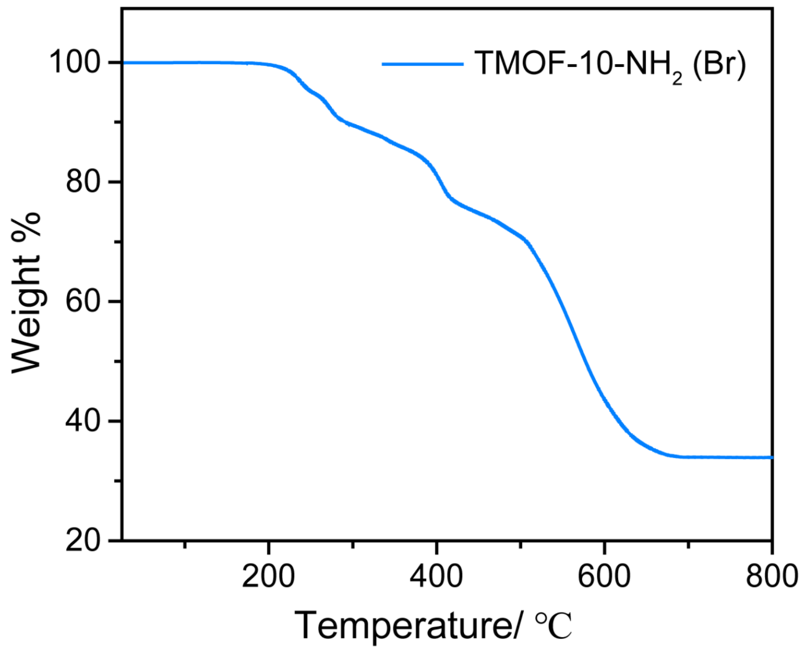


**Supplementary Figure 5 | TGA of TMOF-10-NH$_2$(Br).** Thermogravimetric analysis of TMOF-10-NH$_2$(Br) in $N_2$ flow.

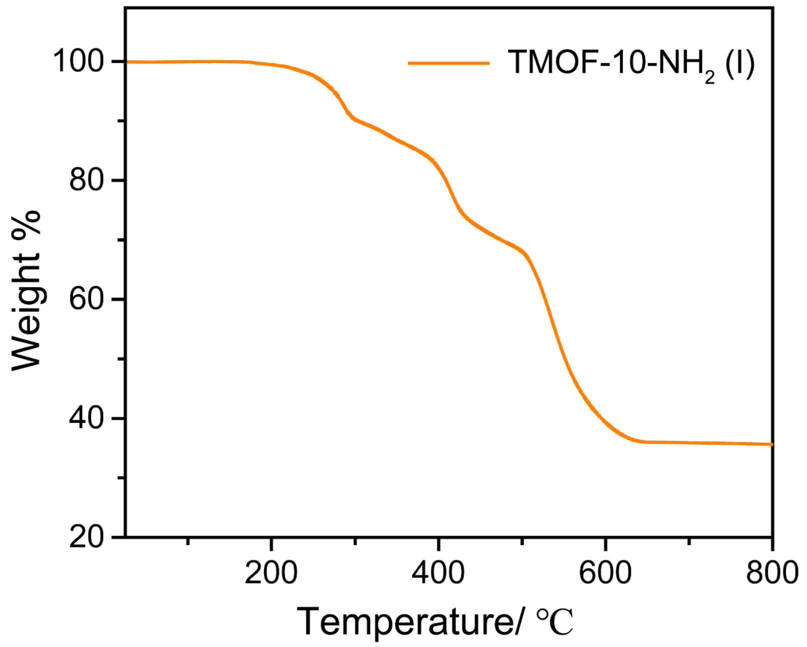


**Supplementary Figure 6 | TGA of TMOF-10-NH$_2$(I).** Thermogravimetric analysis of TMOF-10-NH$_2$(I) in $N_2$ flow.

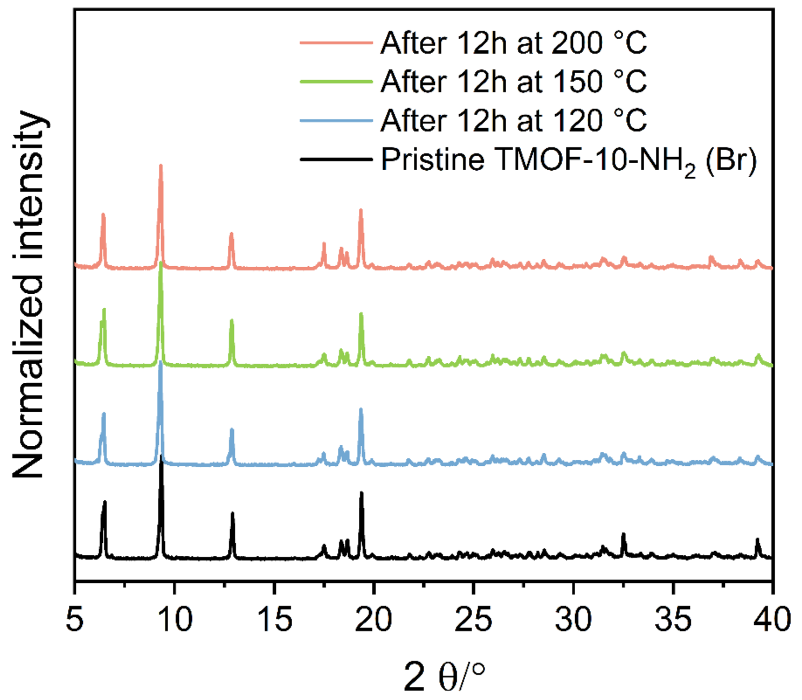


**Supplementary Figure 7 | Thermal stability of TMOF-10-$NH_2$(Br).** PXRD patterns of TMOF-10-$NH_2$(Br) before and after thermal treatment in air for 12 h.

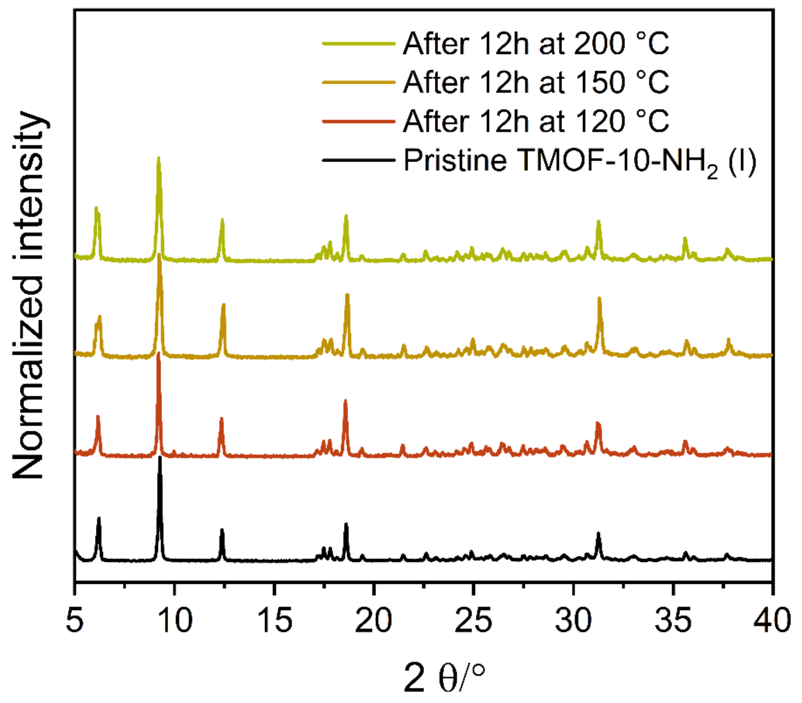


**Supplementary Figure 8 | Thermal stability of TMOF-10-$NH_2$(I).** PXRD patterns of TMOF-10-$NH_2$(I) before and after thermal treatment in air for 12 h.

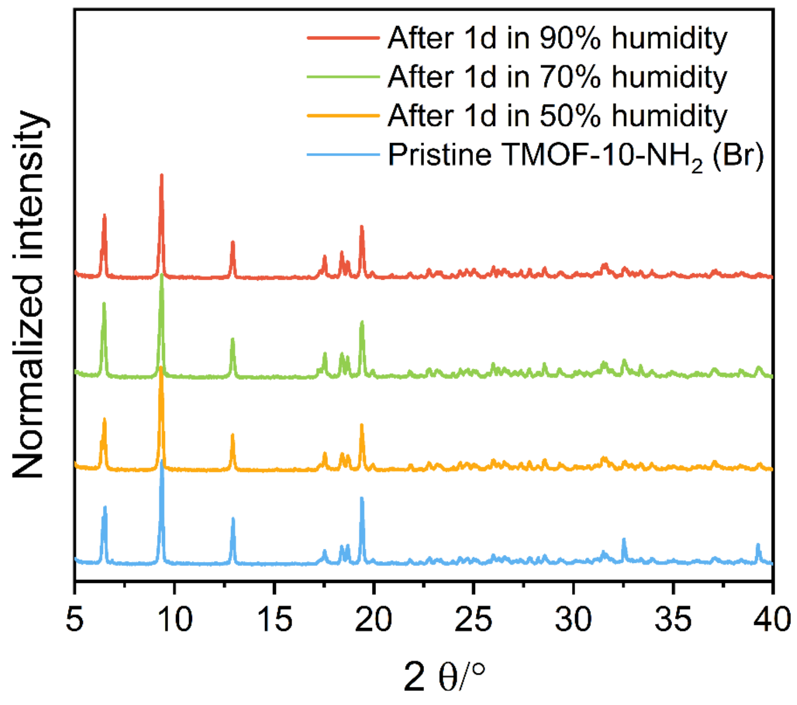


**Supplementary Figure 9 | Moisture stability of TMOF-10-NH$_2$(Br).** PXRD patterns of TMOF-10-NH$_2$(Br) before and after 1d treatment in different relative humidity.

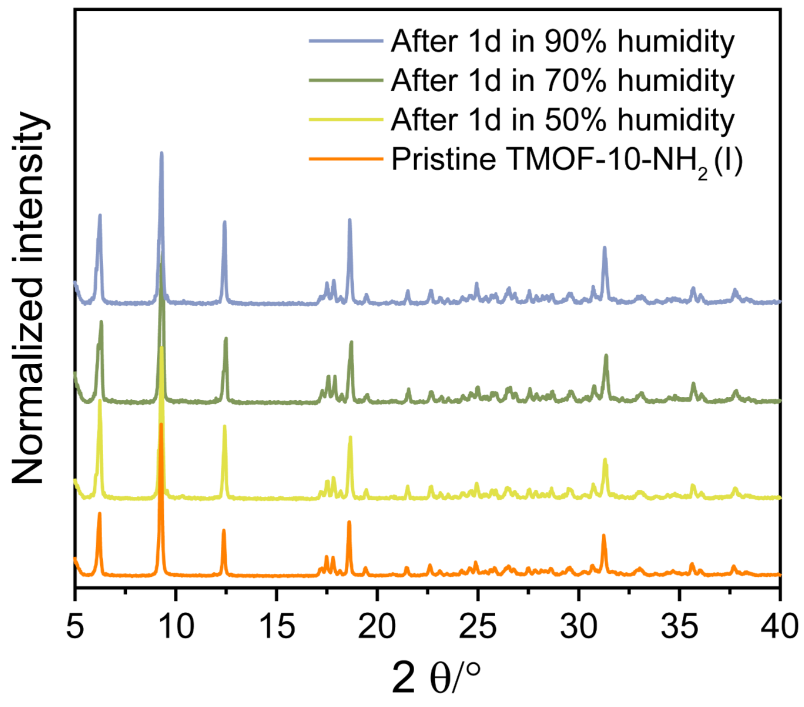


**Supplementary Figure 10 | Moisture stability of TMOF-10-NH$_2$(I).** PXRD patterns of TMOF-10-NH$_2$(I) before and after 1d treatment in different relative humidity.

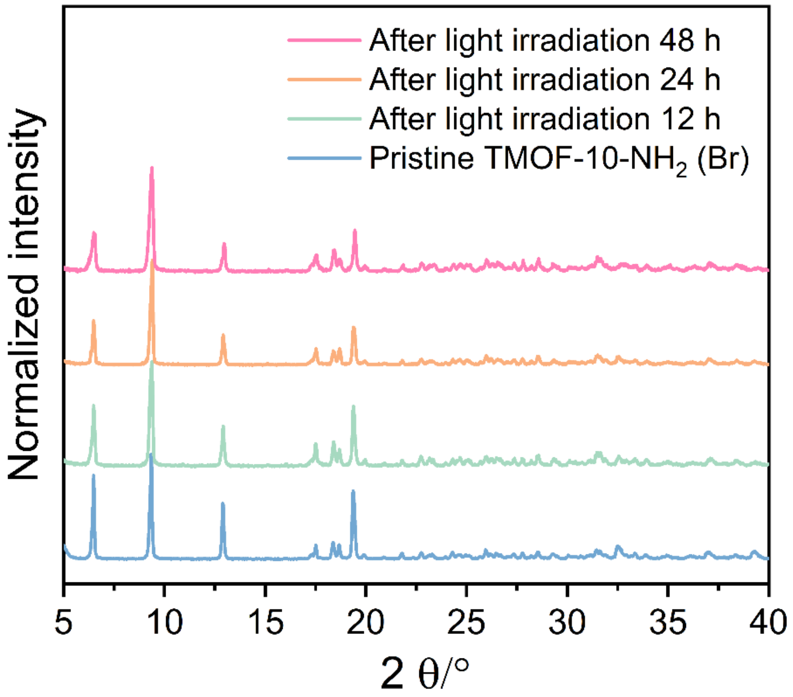


**Supplementary Figure 11 | Photostability of TMOF-10-NH$_2$(Br).** PXRD patterns of TMOF-10-NH$_2$(Br) before and after irradiation by a 300 W Xenon lamp (24 W/cm$^2$) at room temperature for a certain period of time.

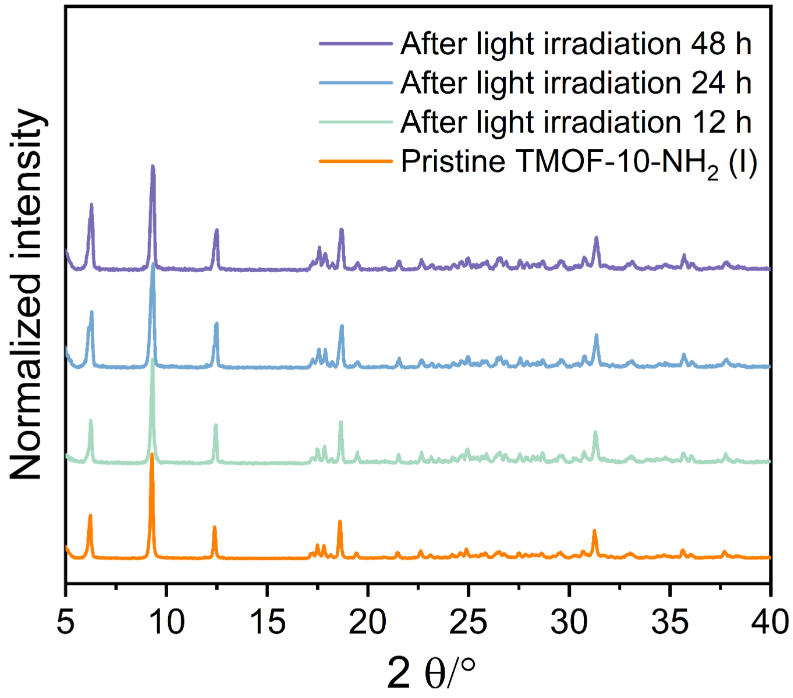


**Supplementary Figure 12 | Photostability of TMOF-10-NH$_2$(I).** PXRD patterns of TMOF-10-NH$_2$(I) before and after irradiation by a 300 W Xenon lamp (24 W/cm$^2$) at room temperature for a certain period of time.

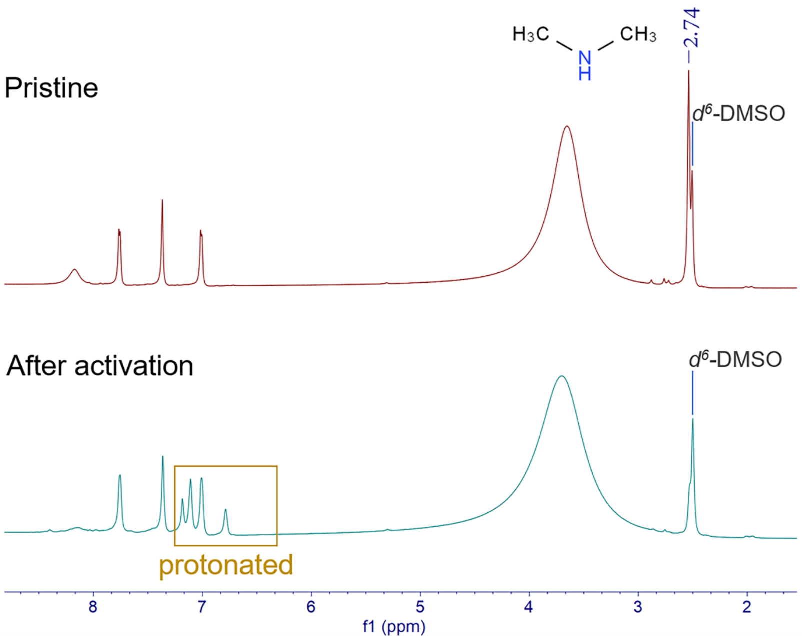


**Supplementary Figure 13 | Guest exchange of TMOF-10-$NH_2$(I).** $^1$H-NMR spectra of diluted HF/$d^6$-DMSO-digested TMOF-10-$NH_2$(I) (before and after activation).

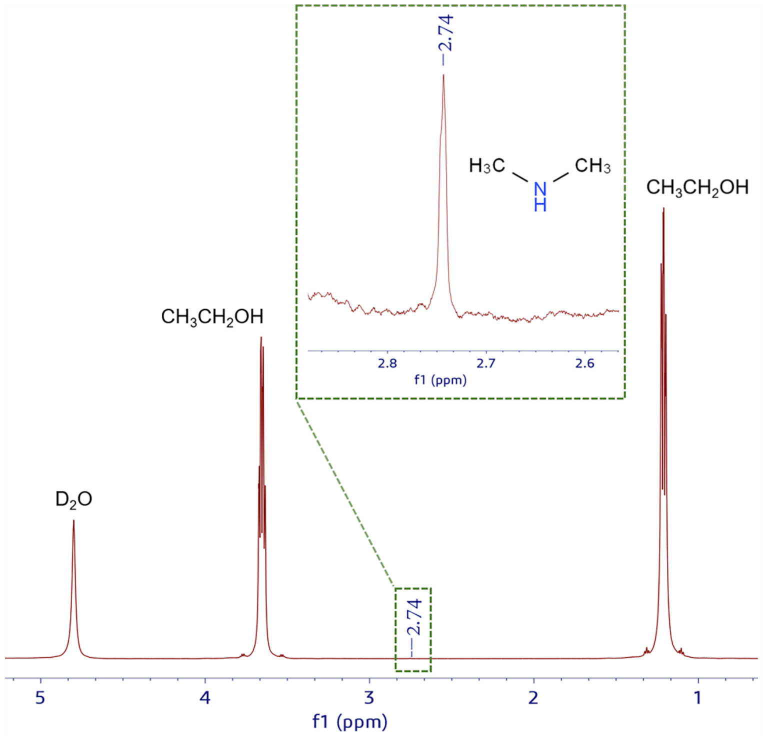


**Supplementary Figure 14 | $^1$H-NMR spectra of guest exchange solution.** $^1$H-NMR spectra of EtOH supernatant after activation of TMOF-10-$NH_2$(I).

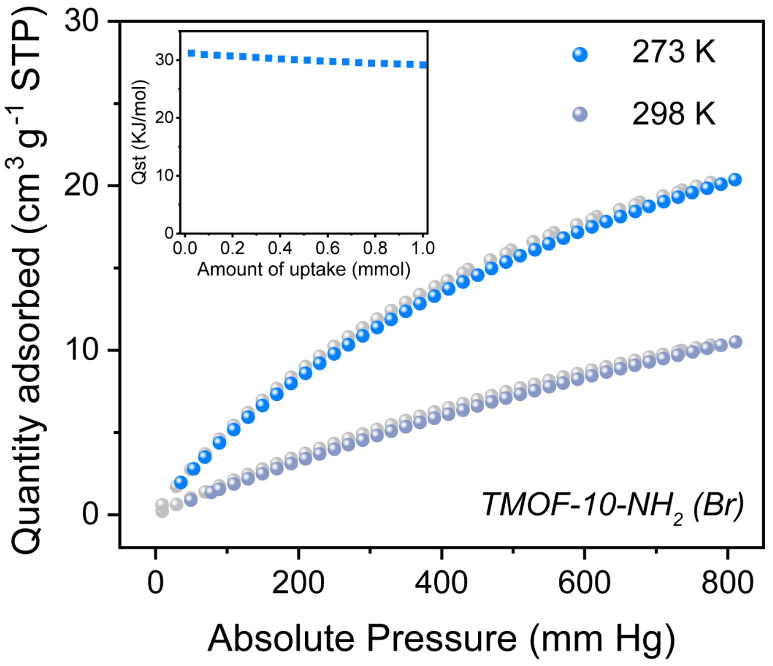


**Supplementary Figure 15 | $CO_2$ sorption of TMOF-10-$NH_2$(Br).** $CO_2$ absorption isotherms of TMOF-10-$NH_2$(Br) at 273 K and 298 K. The inset is the Qst of $CO_2$ sorption.

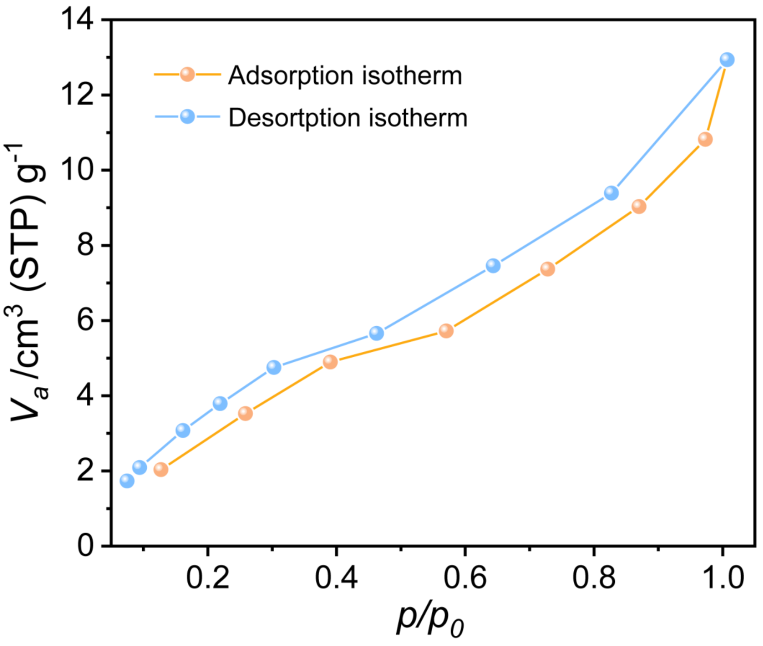


**Supplementary Figure 16 | $H_2O$ vapor sorption of TMOF-10-$NH_2$(I).** $H_2O$ vapor sorption isotherm of TMOF-10-$NH_2$(I) at 298 K.

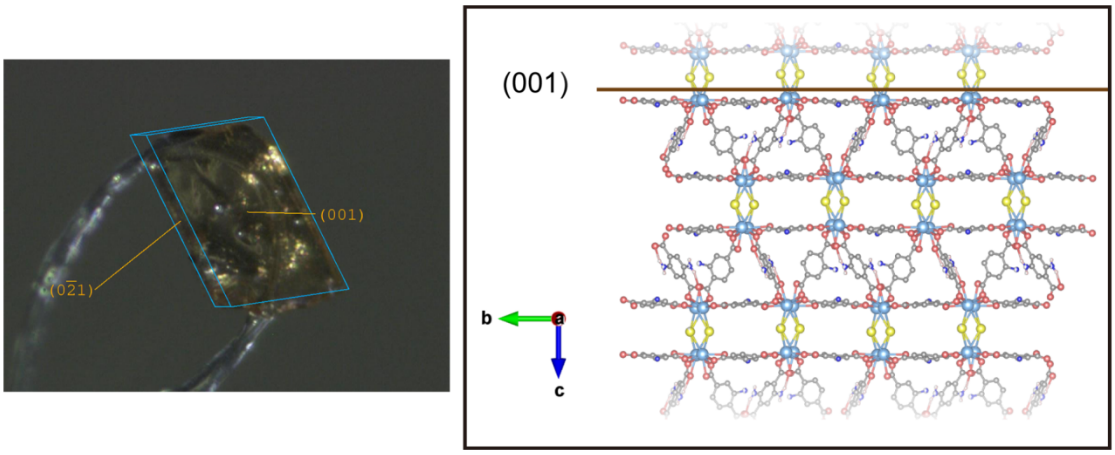


**Supplementary Figure 17 | Characterization of crystal facets.** Determination of exposed crystal facets of a TMOF-10-$NH_2$(I) single crystal.

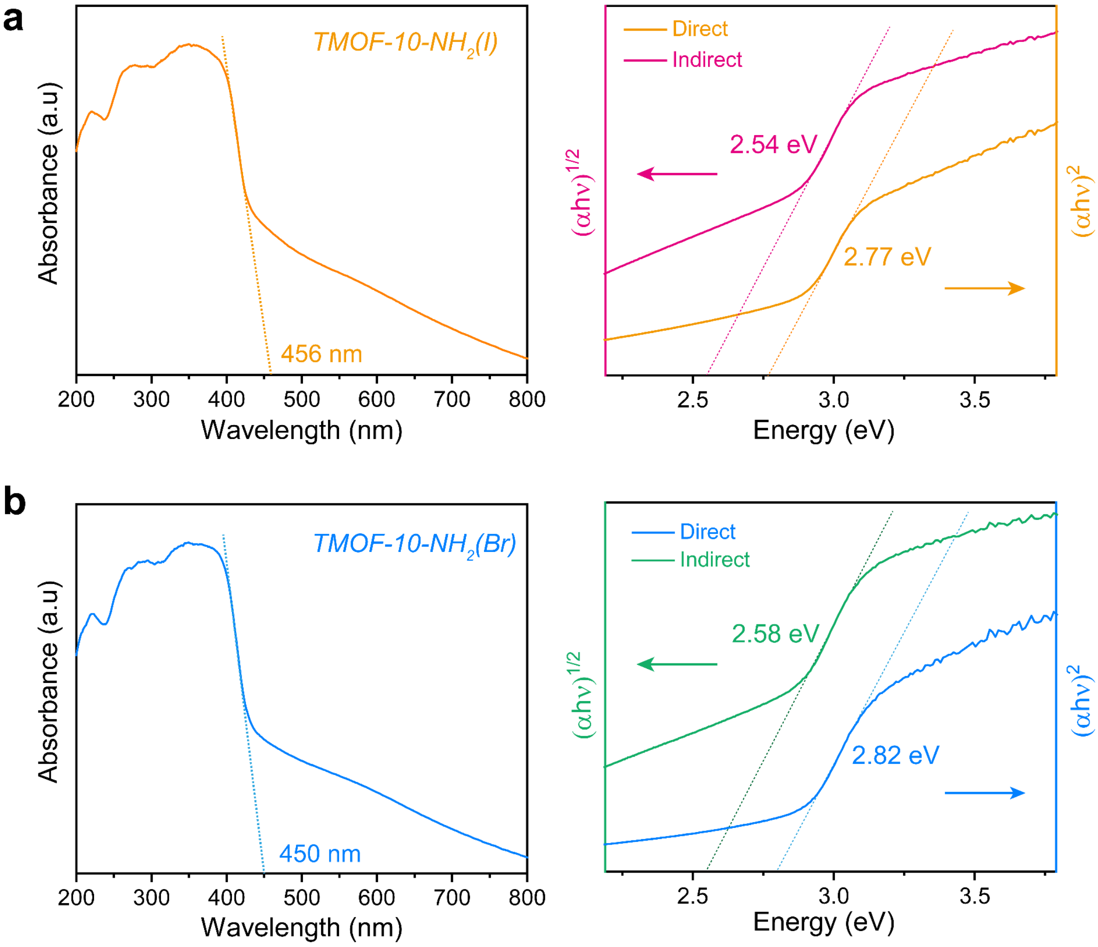


**Supplementary Figure 18 | Band structure studies.** UV-Vis diffuse reflectance spectra (DRS) and Tauc plots of TMOF-10-$NH_2$(I) (a) and TMOF-10-$NH_2$(Br) (b), respectively.

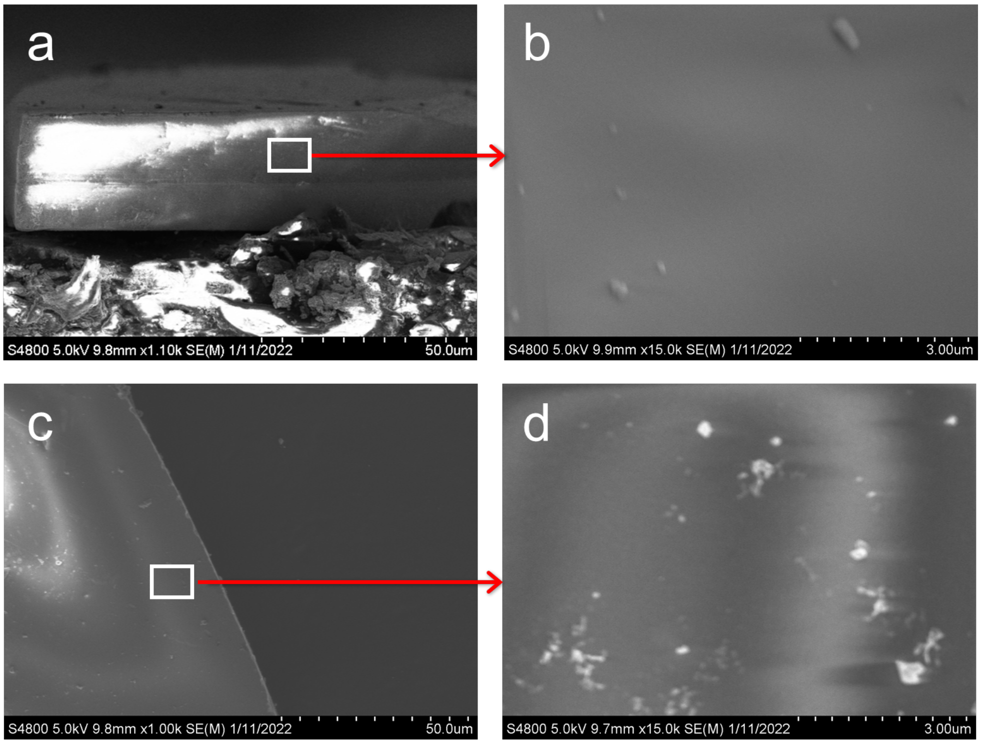


**Supplementary Figure 19 | SEM images of TMOF-10-NH$_2$(I). a, c,** Cross-sectional and surface SEM images of a TMOF-10-NH$_2$(I) single crystal. No apparent grain boundaries were found on the surface of the TMOF-10-NH$_2$(I) crystal. The dust in **b**, **d** indicate the good focus of electron beam.

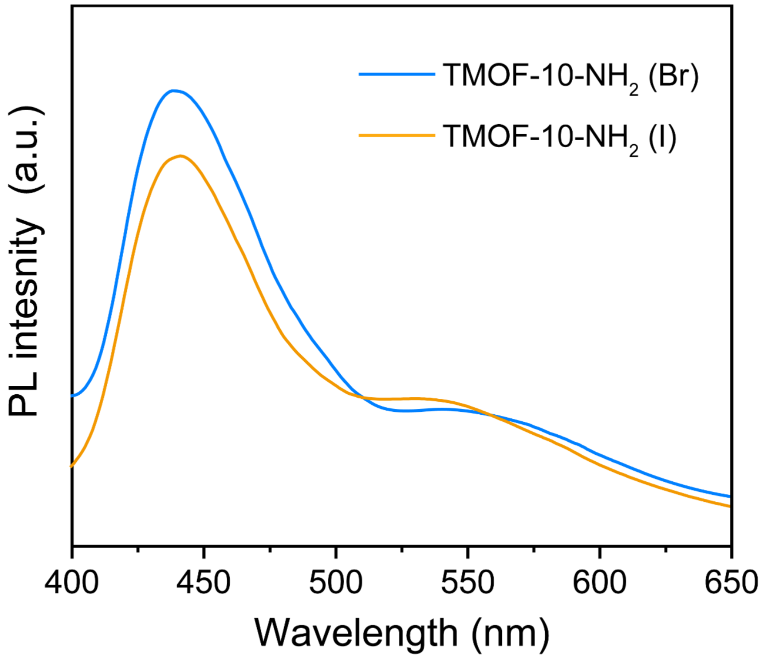


**Supplementary Figure 20 | Photoluminescence (PL) spectra of TMOF-10-NH$_2$.** Steady-state PL spectra of TMOF-10-NH$_2$(I) (yellow) and TMOF-10-NH$_2$(Br) (blue).

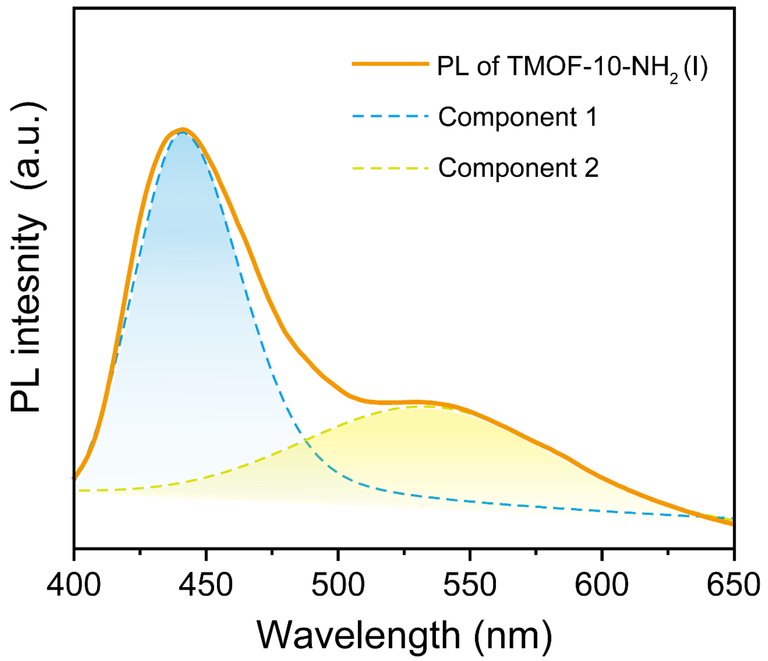


**Supplementary Figure 21 | PL spectra of TMOF-10-$NH_2$(I). A** fit of two PL centers in TMOF-10-$NH_2$(I).

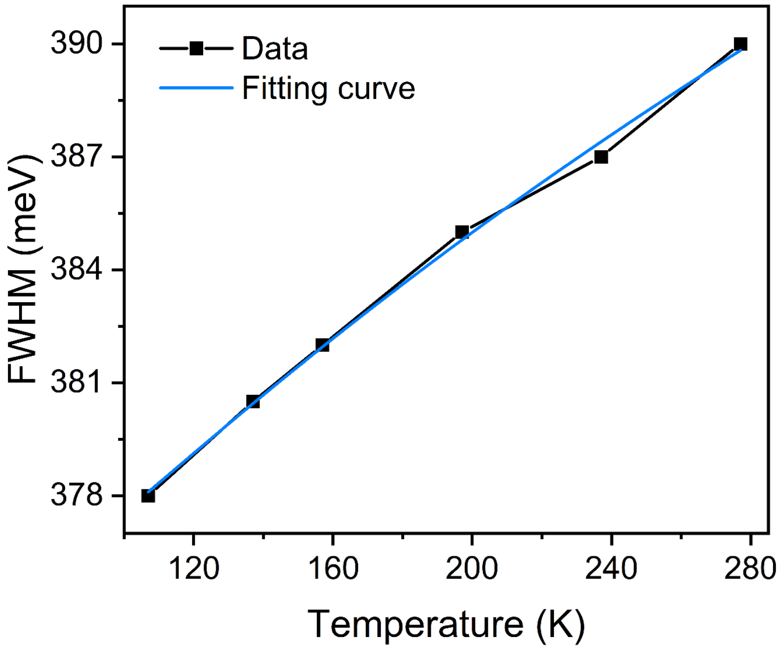


**Supplementary Figure 22 | Temperature-dependent PL spectra of TMOF-10-$NH_2$(I).** Temperature dependence of the self-trapped emission bandwidth (excluding the high-energy shoulder) in TMOF-10-$NH_2$(I) (black) and the best fit (blue) to a model (Eq. 1 in manuscript). The best fit to the data gives a considerable contribution of the electron-phonon coupling [$\Gamma_{LO}$ of 129(10) meV] to the broadening of PL and phonon energy of 18(2) meV.

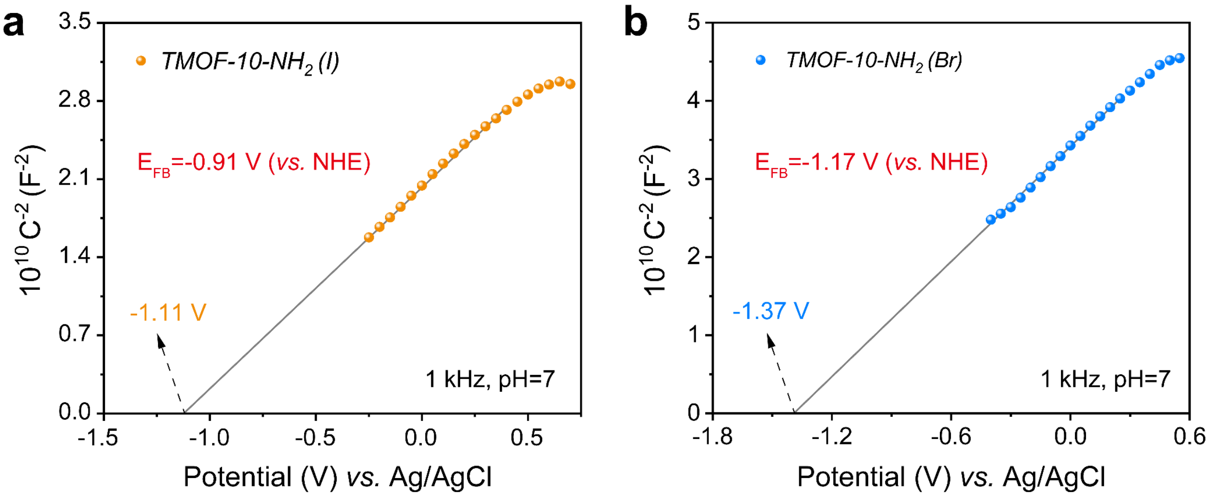


**Supplementary Figure 23 | Mott−Schottky plots of TMOF-10-NH$_2$. a,** The Mott−Schottky profile for TMOF-10-NH$_2$(I); **b,** The Mott−Schottky profile for TMOF-10-NH$_2$(Br). $E_{FB}$ represents the flat band potential.

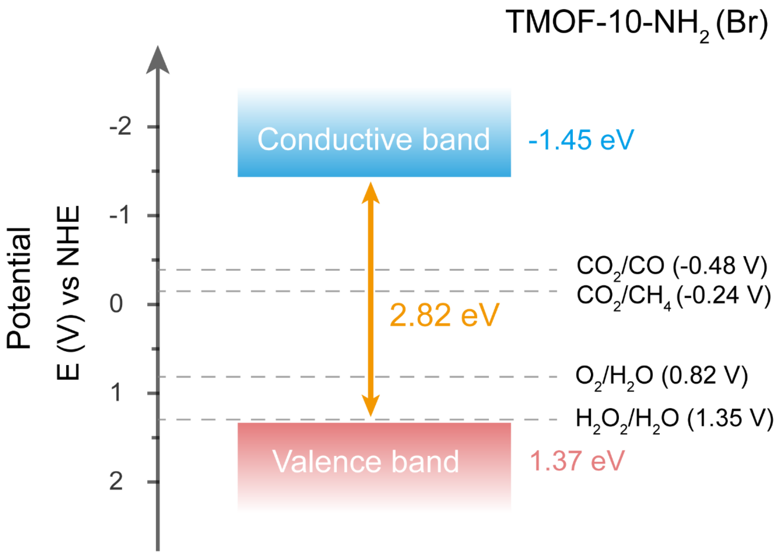


**Supplementary Figure 24 | Band alignment of TMOF-10-NH$_2$(Br).** Schematics illustrating the band structures of TMOF-10-NH$_2$(Br). Yellow arrow represents the the width of the bandgap.

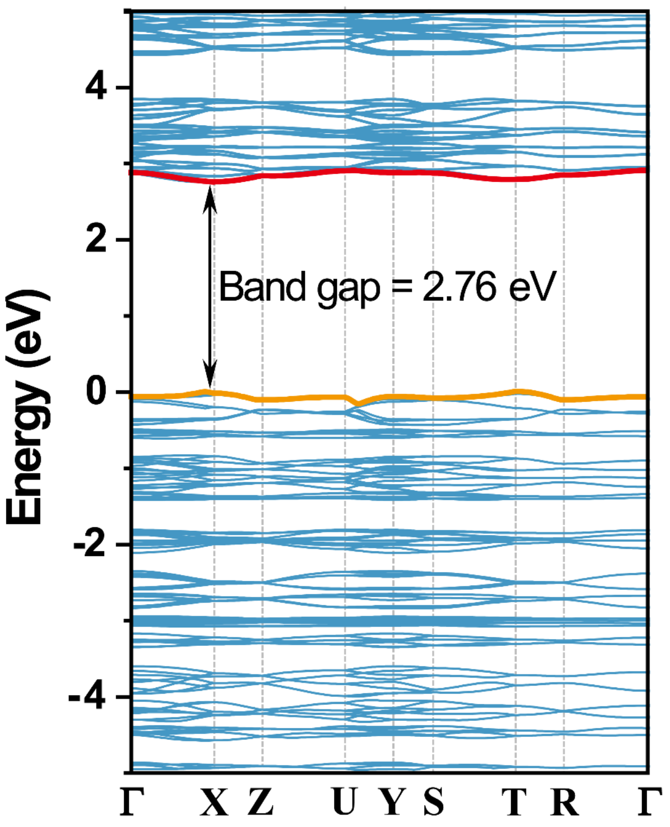


**Supplementary Figure 25 | Band structure of TMOF-10-$NH_2$(I).** DFT calculations: band structure of TMOF-10-$NH_2$(I).

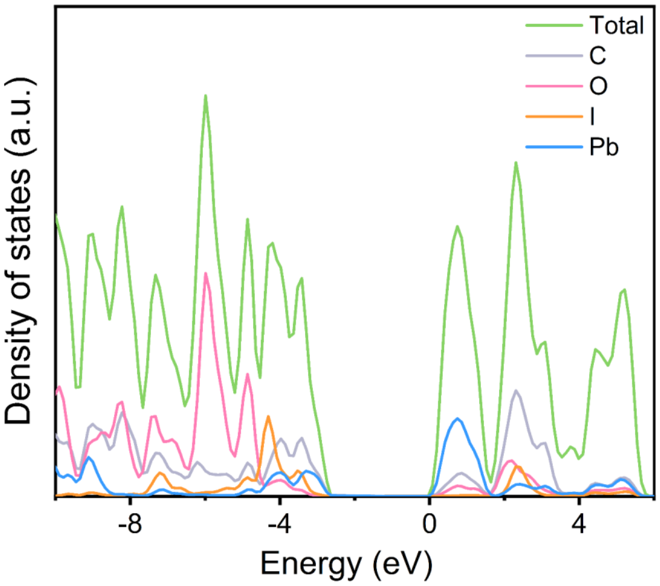


**Supplementary Figure 26 | Total density of states of TMOF-10-$NH_2$(I).** DFT calculations: the total density of states of TMOF-10-$NH_2$(I).

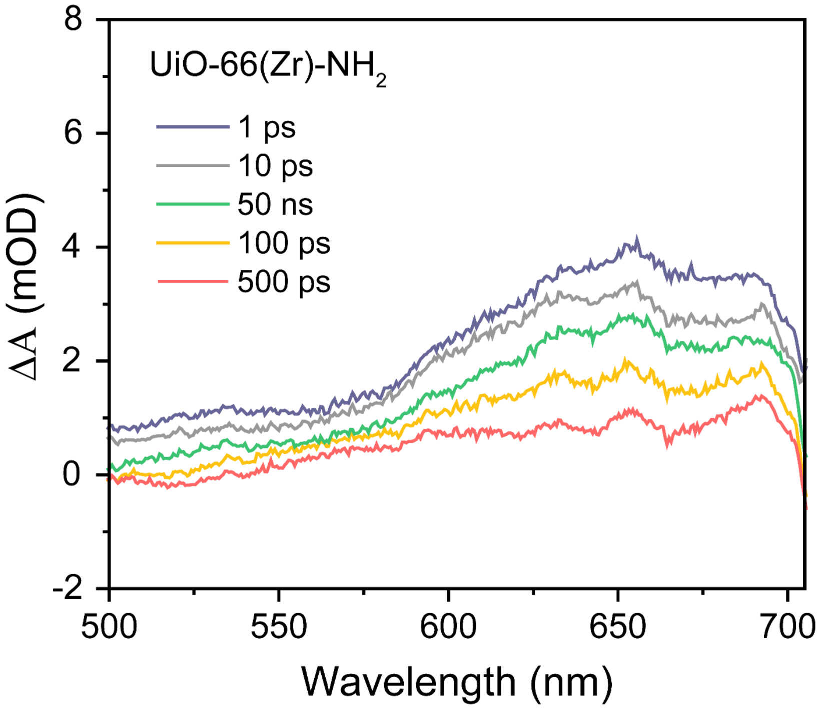


**Supplementary Figure 27 | TA spectra of UiO-66(Zr)-NH$_2$.** Femtosecond ultrafast TA spectra of UiO-66(Zr)-NH$_2$ at different time delays under 385 nm excitation.

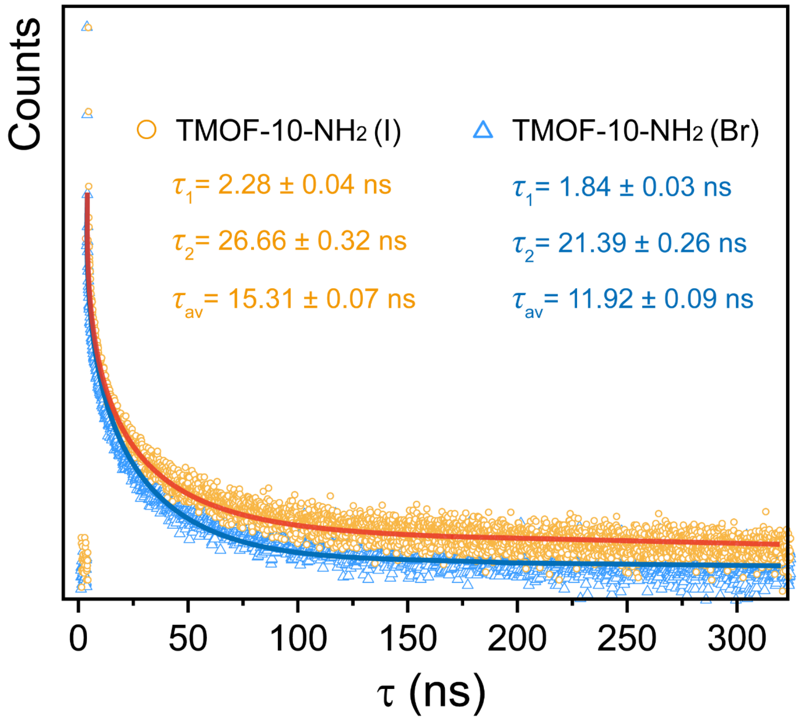


**Supplementary Figure 28 | PL lifetime of TMOF-10-NH$_2$.** PL decay and the biexponential fit of TMOF-10-NH$_2$(I) and TMOF-10-NH$_2$(Br) at room temperature.

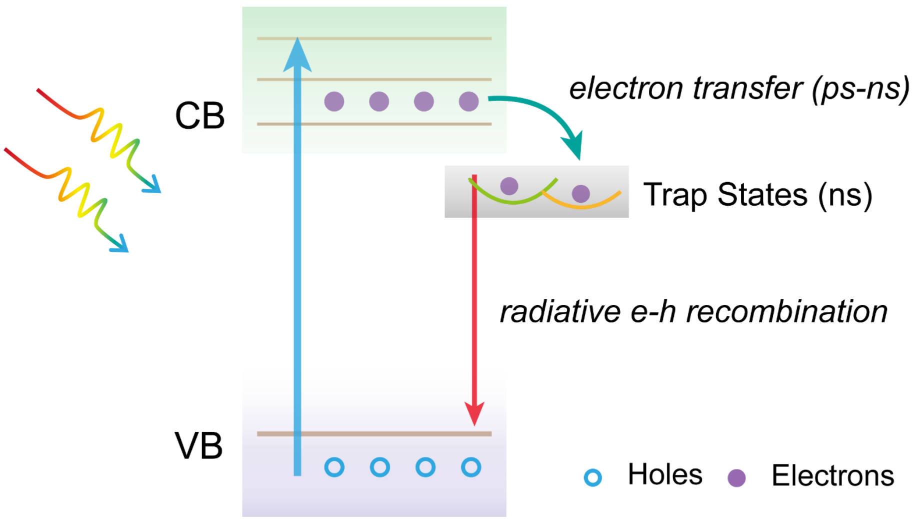


**Supplementary Figure 29 | Schematics illustrating the photoexcited dynamics involved in TMOF-10-$NH_2$(I).** Possible photoexcitation dynamics in TMOF-10-$NH_2$(I) based on TA and transient-state PL studies. Arrows represent the electron transfer processes. VB, valence band; CB, conduction band.

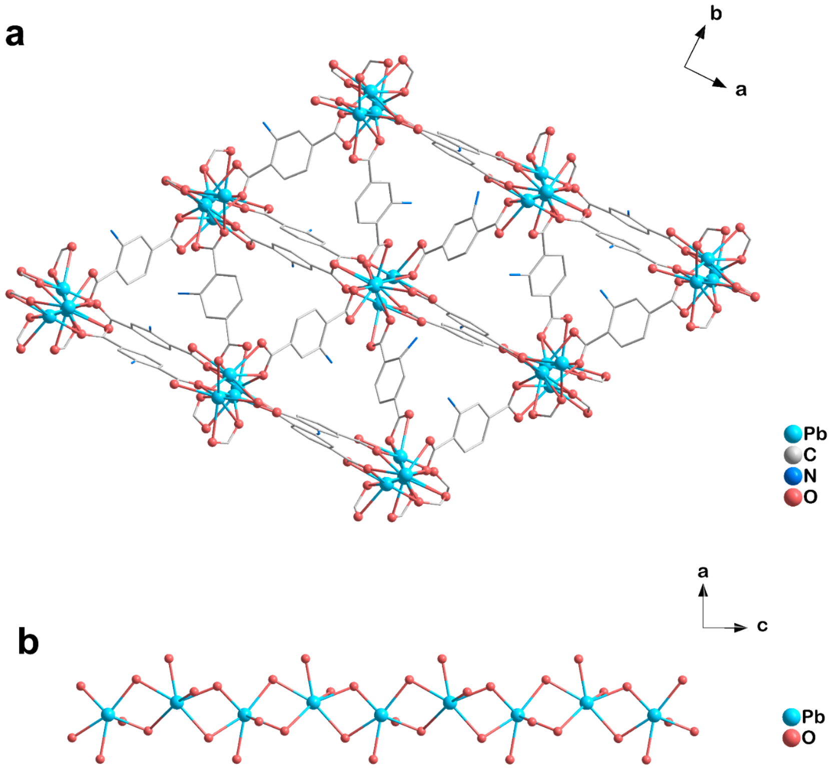


**Supplementary Figure 30 | X-ray crystallographic views of [Pb($NH_2$-bdc)]$_n$ a,** Crystallographic view of [Pb($NH_2$-bdc)]$_n$ along the *c*-axis. **b,** Crystallographic view of a [$PbO_4$] chain along *b*-axis.

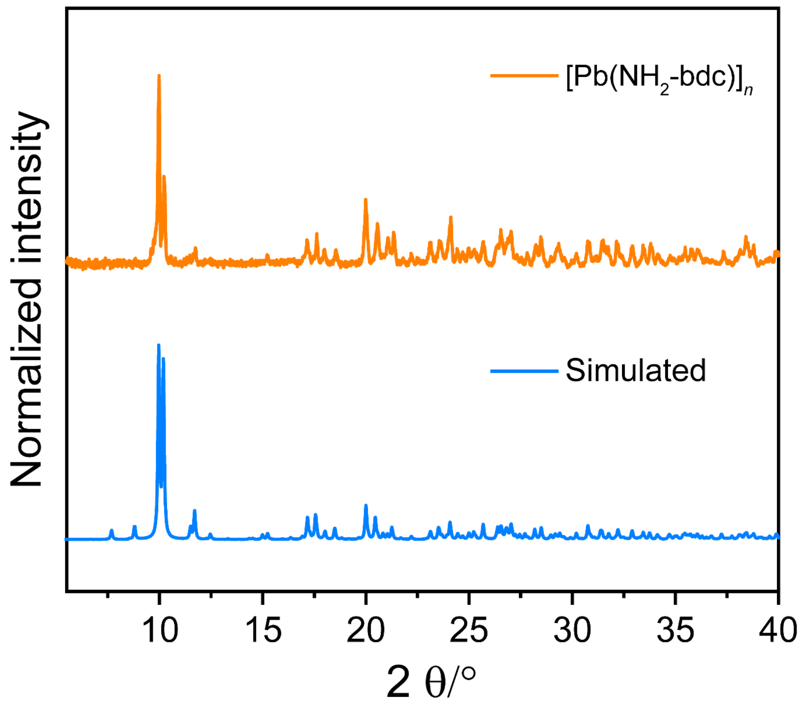


**Supplementary Figure 31 | PXRD of [Pb($NH_2$-bdc)]$_n$.** Experimental and simulated PXRD of [Pb($NH_2$-bdc)]$_n$. The good matching confirms the phase purity of as-synthesized crystalline materials.

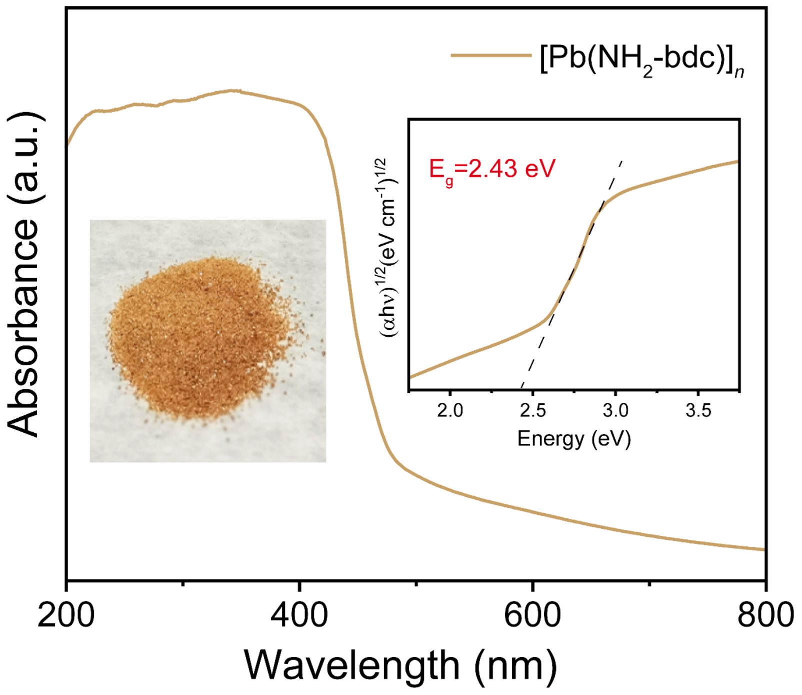


**Supplementary Figure 32 | Photophysical properties of [Pb($NH_2$-bdc)]$_n$.** UV-Vis DRS of as-synthesized [Pb($NH_2$-bdc)]$_n$. The inset is Tauc plot and photoimage of [Pb($NH_2$-bdc)]$_n$.

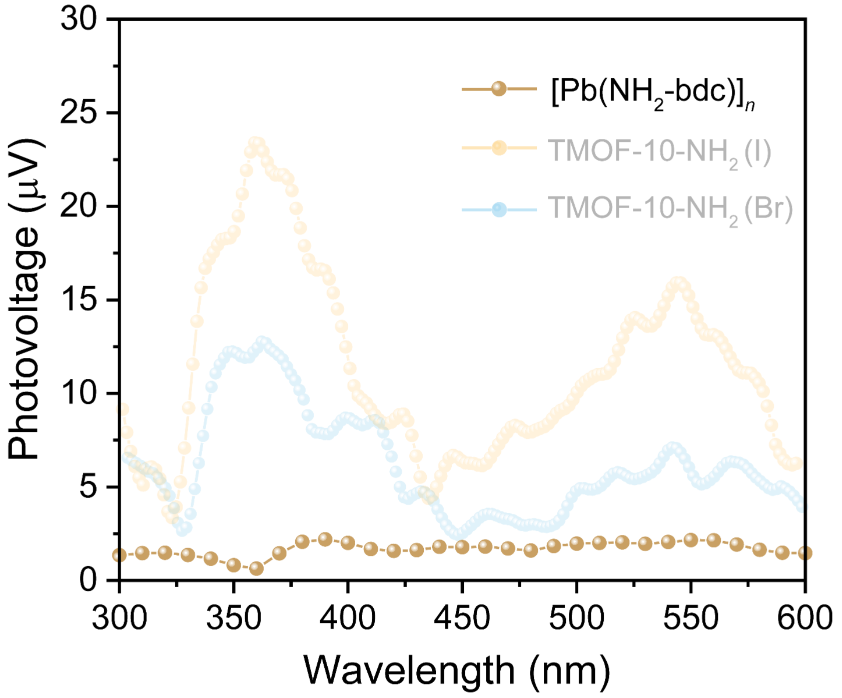


**Supplementary Figure 33 | SPV measurement of [Pb($NH_2$-bdc)]*n*.** Comparison of SPV spectra of [Pb($NH_2$-bdc)]*n* and TMOF-10-$NH_2$.

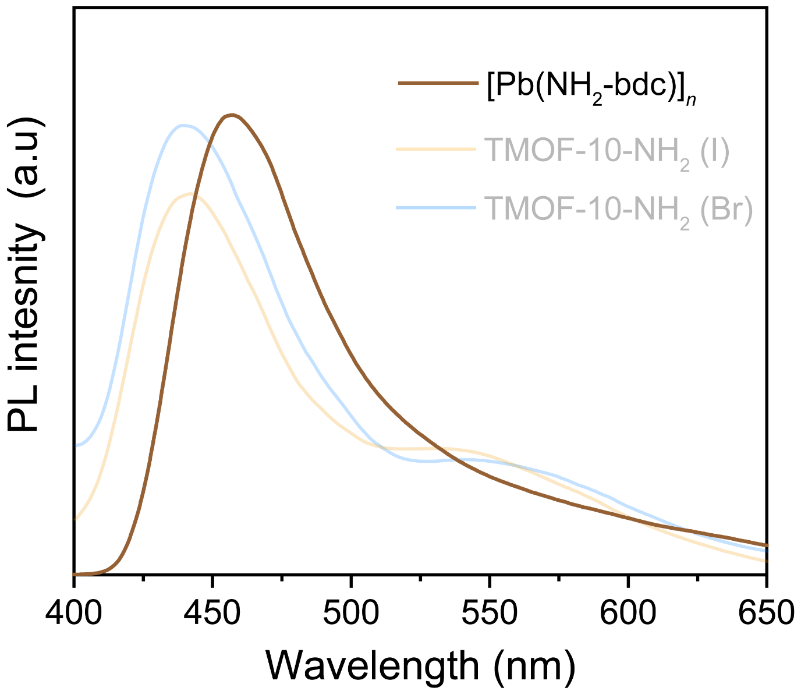


**Supplementary Figure 34 | PL spectra of [Pb($NH_2$-bdc)]*n*.** Comparison of PL spectra of [Pb($NH_2$-bdc)]*n* and TMOF-10-$NH_2$.

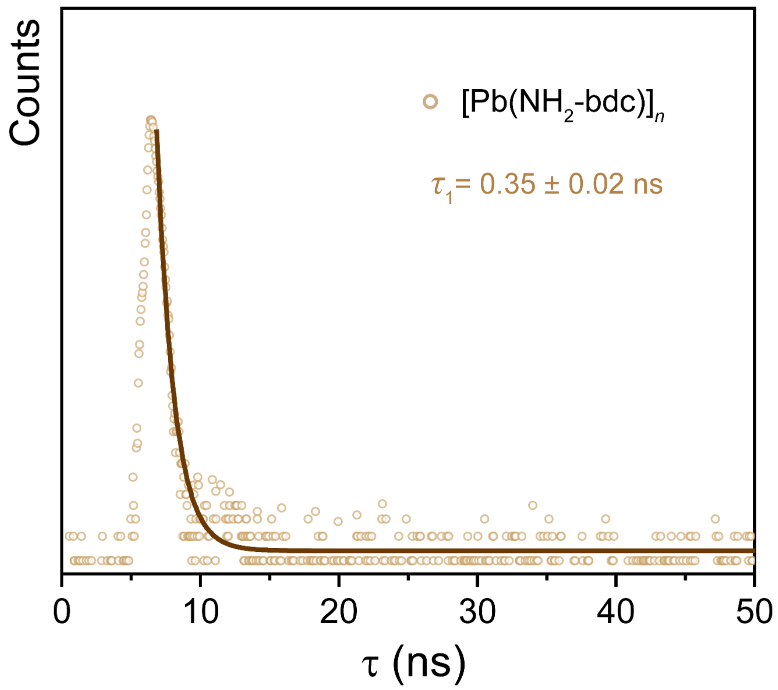


**Supplementary Figure 35 | PL lifetime of $[Pb(NH_2\text{-bdc})]_n$.** PL decay and exponential fitting of $[Pb(NH_2\text{-bdc})]_n$ at room temperature.

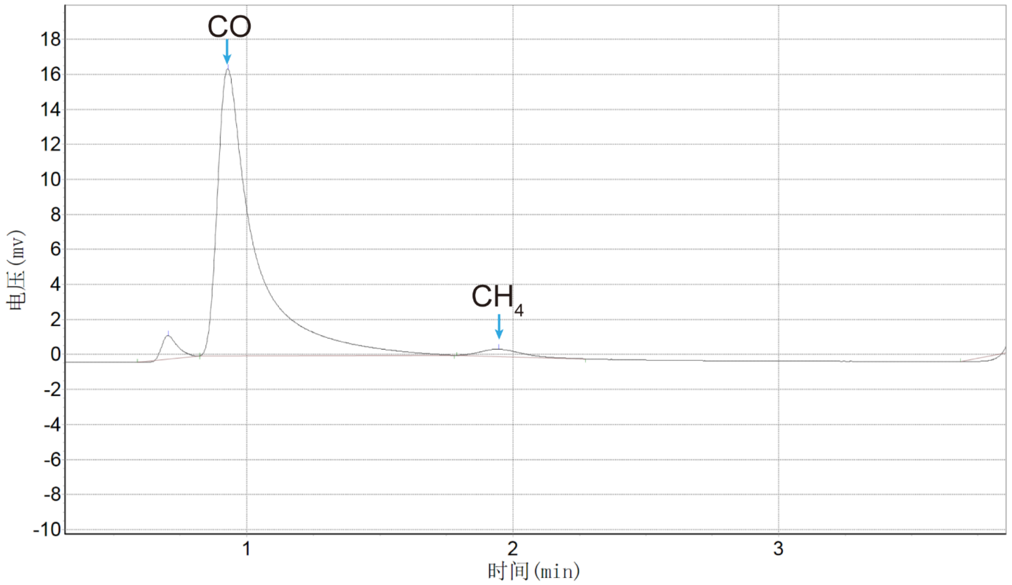


**Supplementary Figure 36 | GC retention time of photoreduced $CO_2$ products.** GC profile of the gaseous products from $CO_2$ photoreduction using TMOF-10-$NH_2$(I) as the photocatalyst.

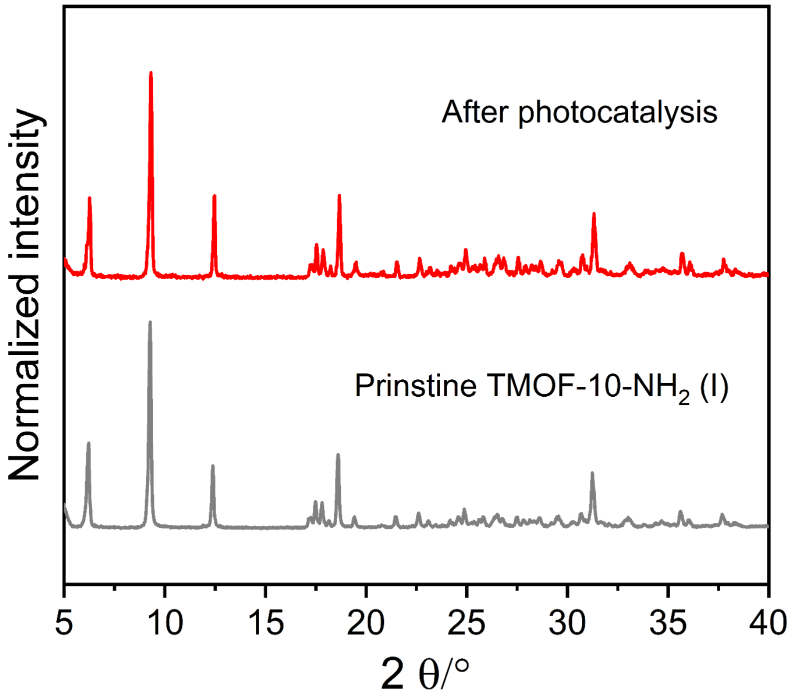


**Supplementary Figure 37 | Photocatalytic stability of TMOF-10-NH$_2$(I).** PXRD patterns of TMOF-10-NH$_2$(I) before and after 24 h photocatalysis reaction.

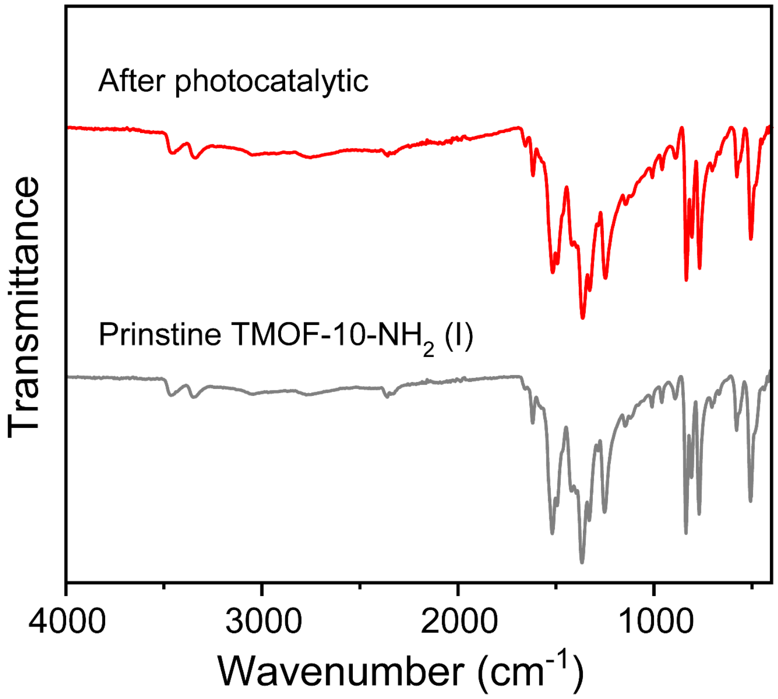


**Supplementary Figure 38 | FT-IR spectra of TMOF-10-NH$_2$(I).** FTIR spectra of TMOF-10-NH$_2$(I) before and after 24 h photocatalysis reaction.

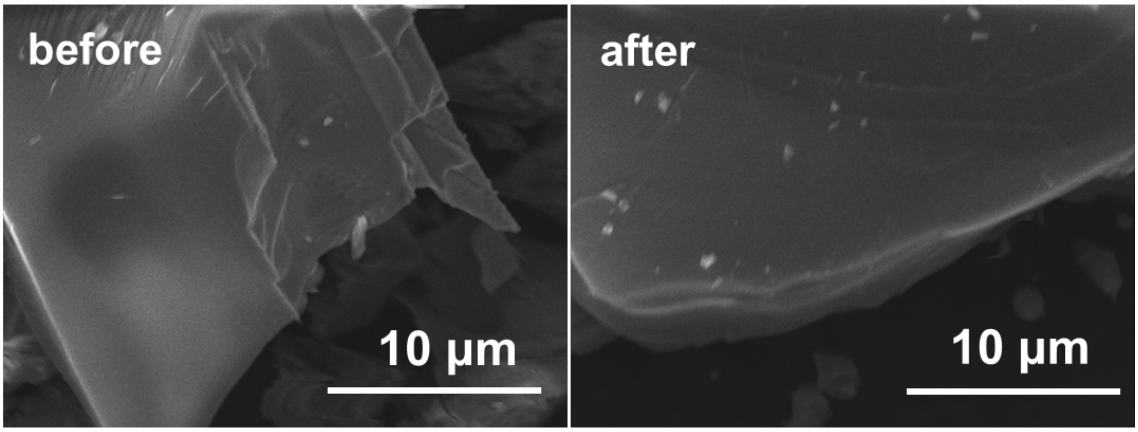


**Supplementary Figure 39 | SEM images of TMOF-10-$NH_2$(I).** SEM images of TMOF-10-$NH_2$(I) before and after 24 h photocatalysis reaction.

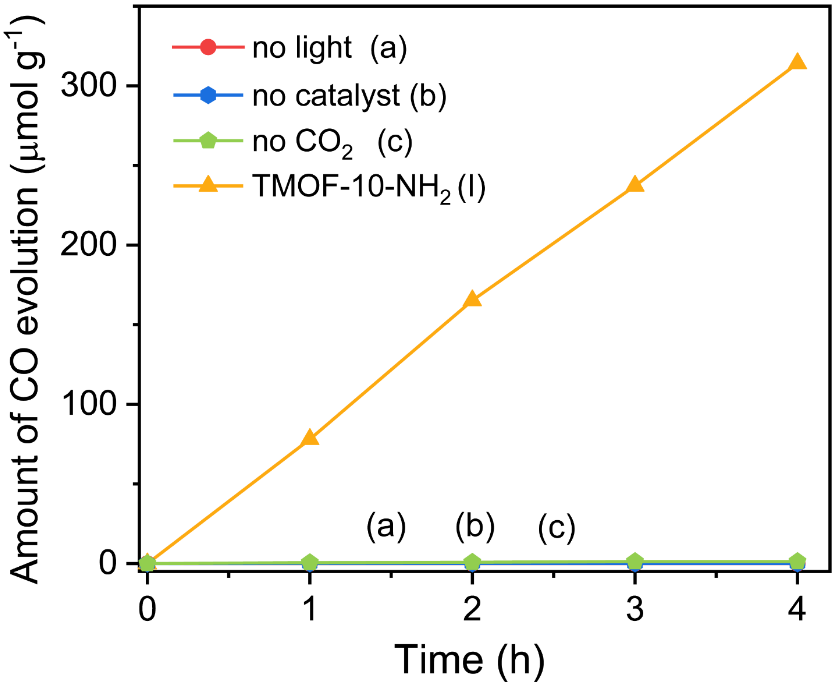


**Supplementary Figure 40 | Control studies for photocatalytic $CO_2$ reduction.** Negligible CO evolution could be detected in experiments without (a) light irradiation, (b) catalyst and (c) $CO_2$.

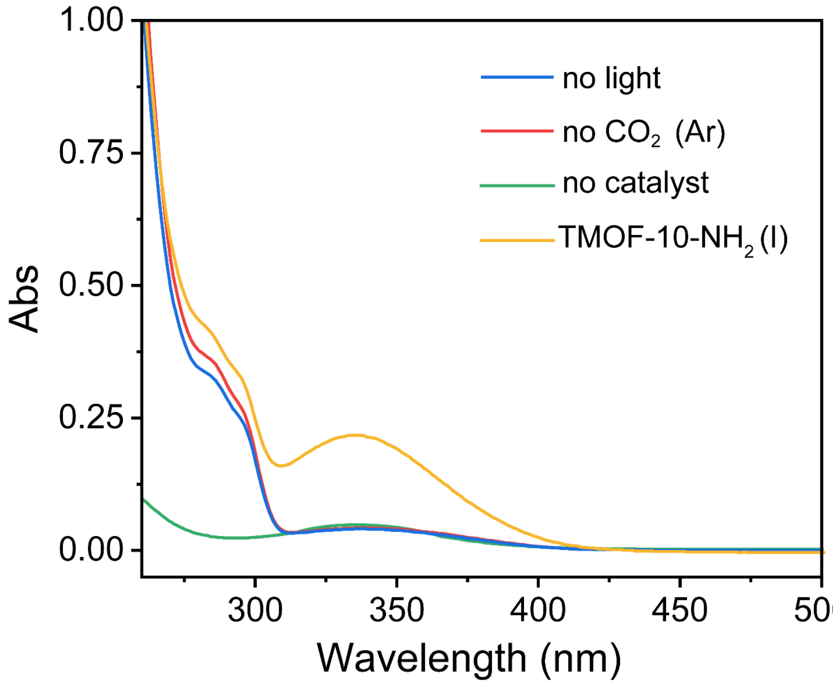


**Supplementary Figure 41 | Control studies for quantitative determination of $H_2O_2$.** Negligible $H_2O_2$ evolution could be detected in experiments without light irradiation, catalyst and $CO_2$ in liquid phase

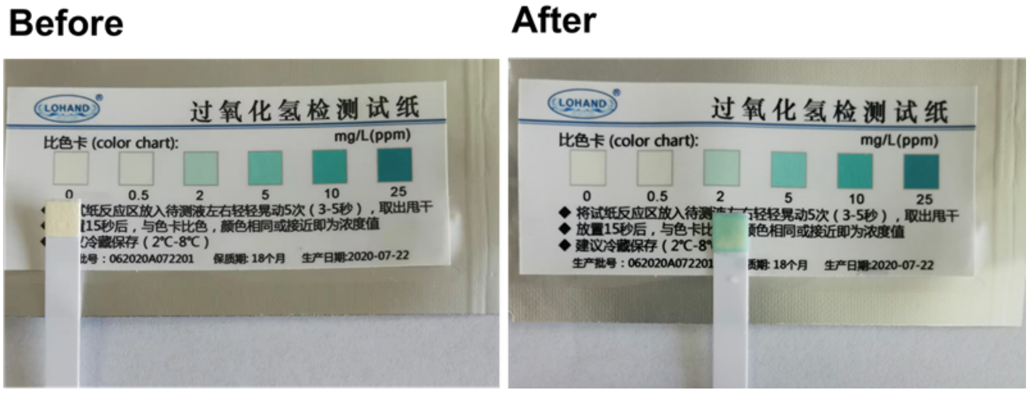


**Supplementary Figure 42 | Quantification of $H_2O_2$ using commercial colorimetric test strips.** Determination of $H_2O_2$ in liquid phase during 12 h photocatalytic $CO_2$ reduction by using of commercial colorimetric test strips.

## Colorimetry test

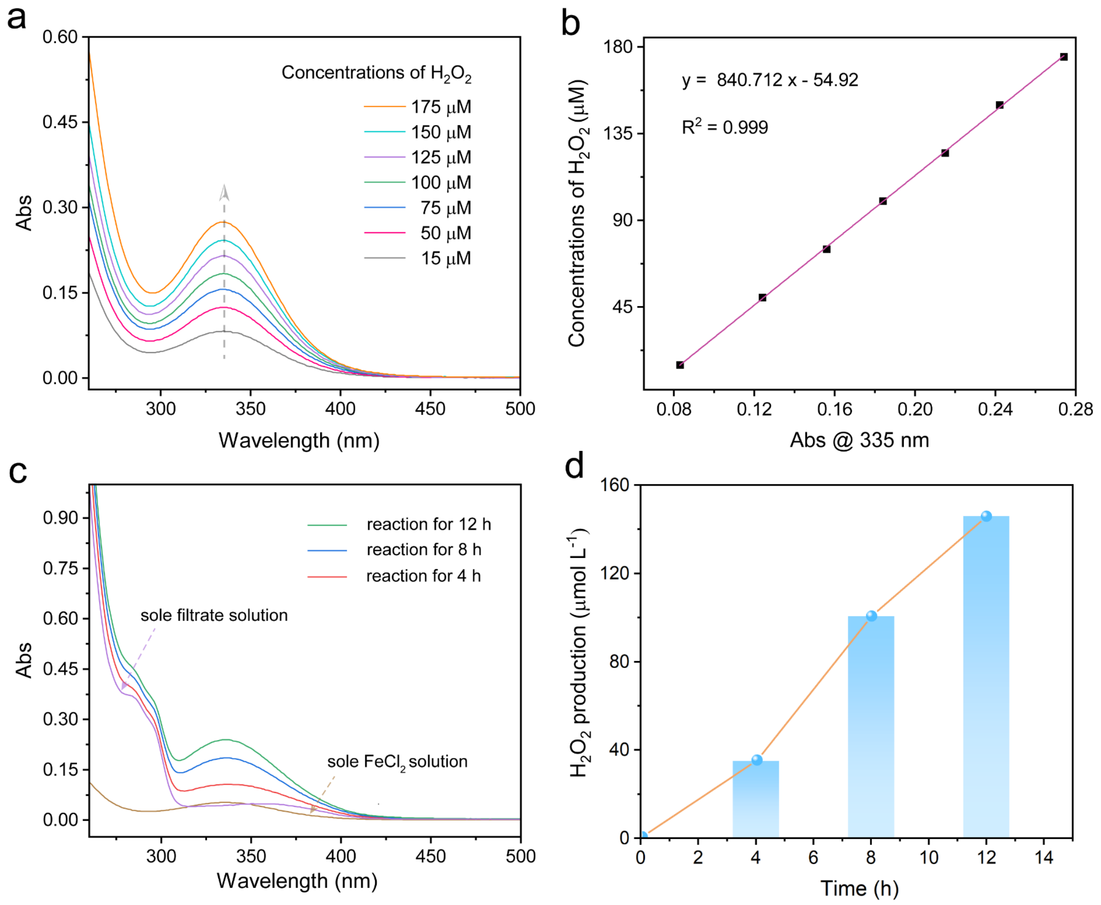


**Supplementary Figure 43 | Quantitative determination of $H_2O_2$ production by colorimetry test. a,** UV-Vis spectra of $H_2O_2$ with certain concentrations in $FeCl_2$/HCl solution. **b,** Linear calibration curve of the net absorbance at 330 nm. **c,** Quantitative determination of $H_2O_2$ product in liquid phase after the 12 h photocatalysis reaction using the colorimetry method. **d,** Time-resolved $H_2O_2$ production determined by the calibration curve.

**DPD/POD method**

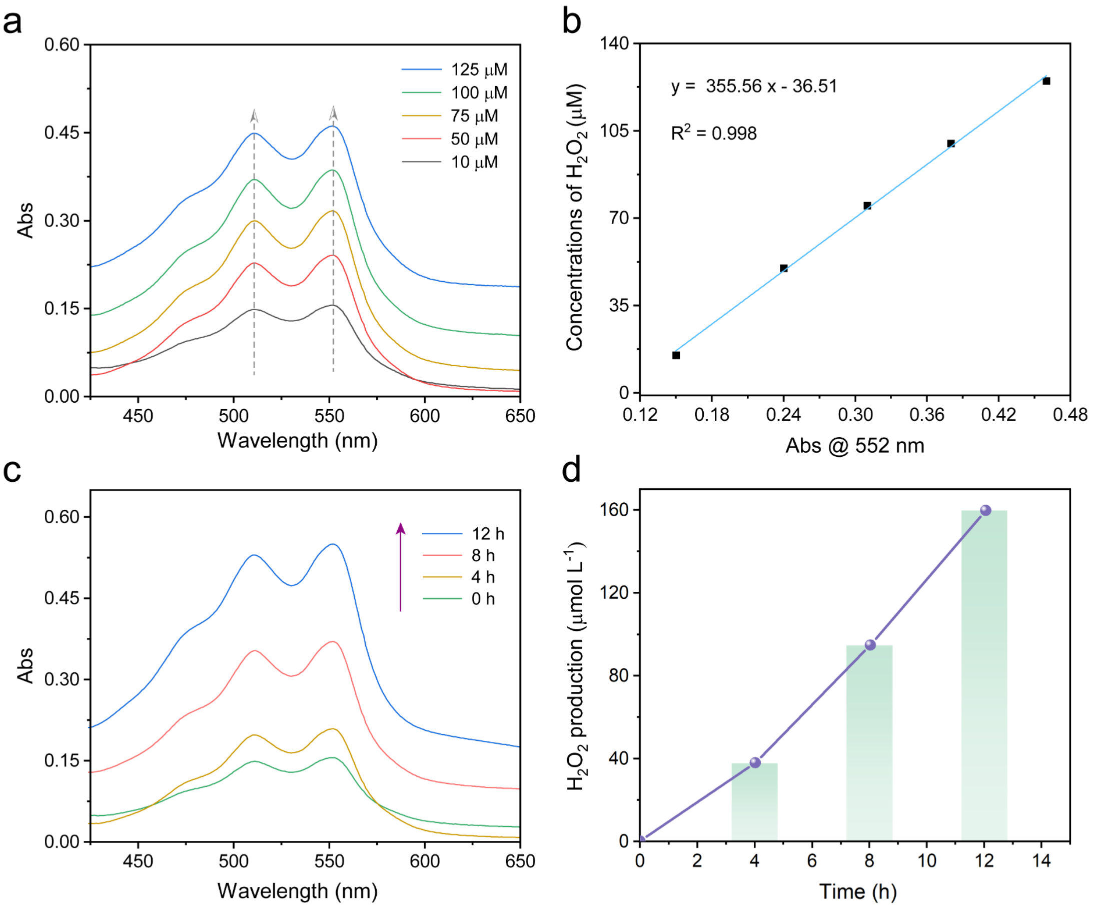


**Supplementary Figure 44 | Quantitative determination of $H_2O_2$ production by DPD/OPD method. a,** UV-Vis spectra of $H_2O_2$ with certain concentrations in DPD/POD solution. **b,** Linear calibration curve of the net absorbance at 552 nm. **c,** Quantitative determination of $H_2O_2$ product in liquid phase after the 12 h photocatalysis reaction using DPD/POD method. **d,** Time-resolved $H_2O_2$ production determined by the calibration curve.

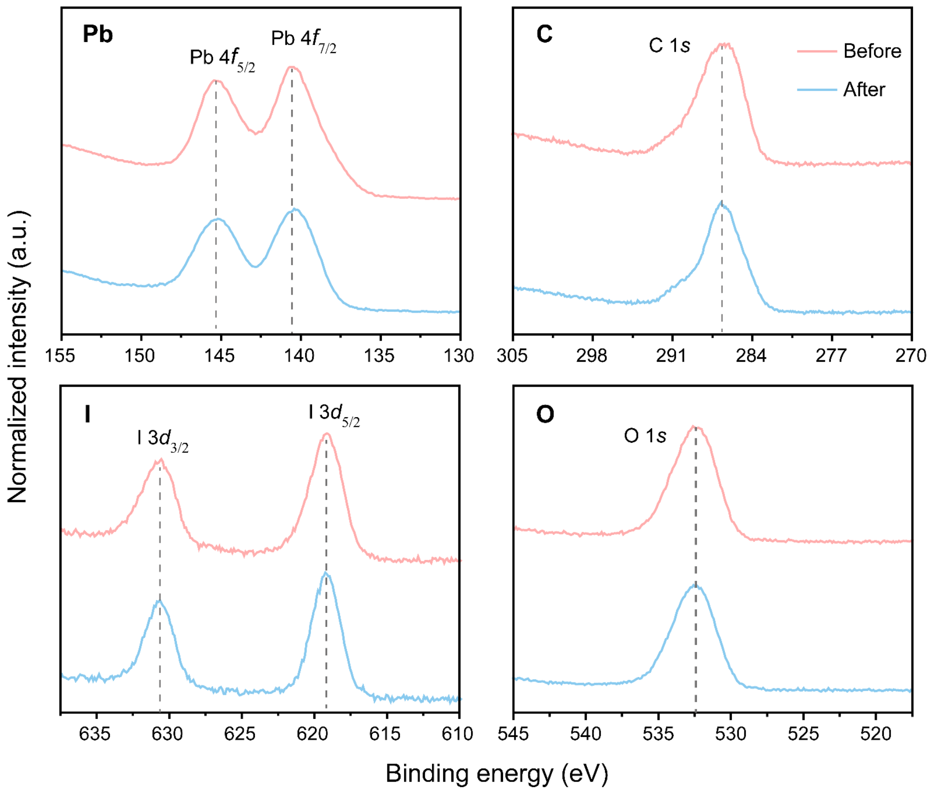


**Supplementary Figure 45 | XPS of TMOF-10-$NH_2$(I).** High-resolution Pb 4*f*, C 1*s*, I 3*d* and O 1*s* XPS spectra of TMOF-10-$NH_2$(I) before and after 12 h photocatalysis reaction.

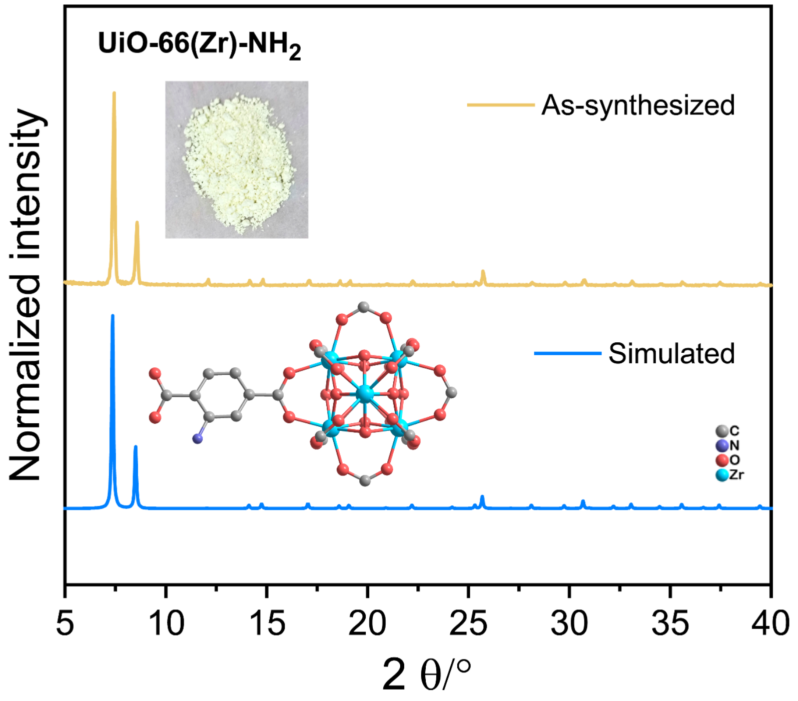


**Supplementary Figure 46 | PXRD of UiO-66(Zr)-$NH_2$.** PXRD pattern of as-synthesized UiO-66(Zr)-$NH_2$ and the simulated pattern from the single-crystal data.

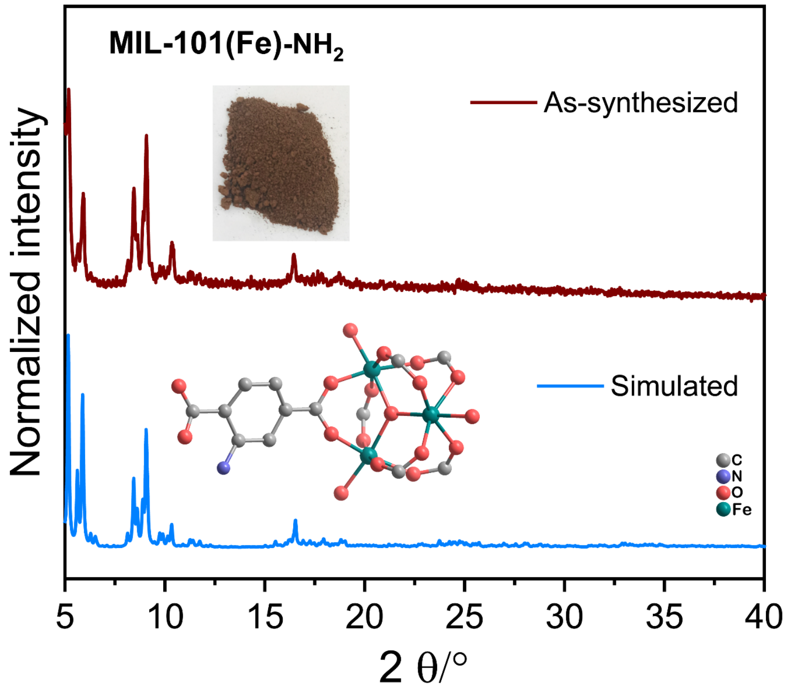


**Supplementary Figure 47 | PXRD of MIL-101(Fe)-$NH_2$.** PXRD pattern of as-synthesized MIL-101(Fe)-$NH_2$ and the simulated pattern from the single-crystal data.

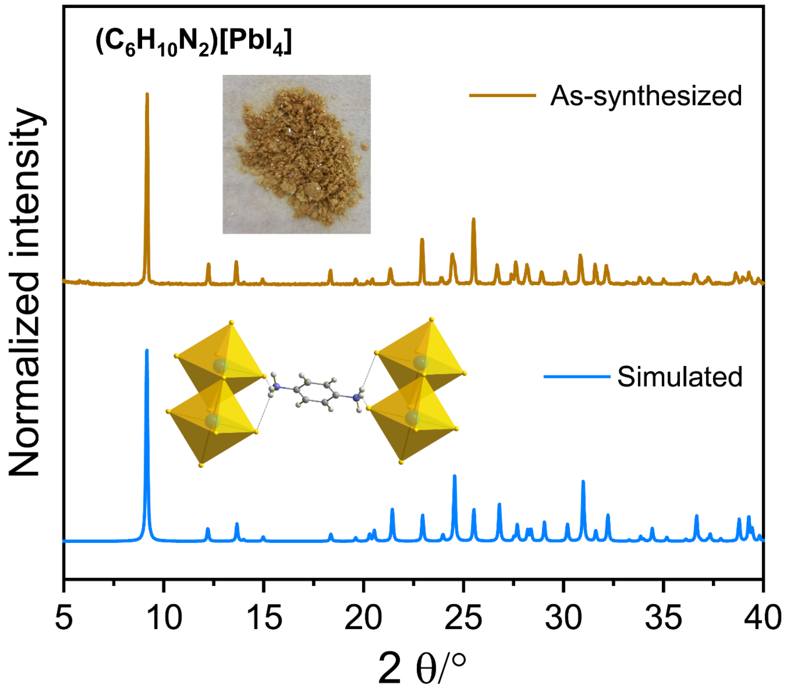


**Supplementary Figure 48 | PXRD of ($C_6H_{10}N_2$)[$PbI_4$].** PXRD pattern of as-synthesized ($C_6H_{10}N_2$)[$PbI_4$] (1D perovskite) and the simulated pattern from the single-crystal data.

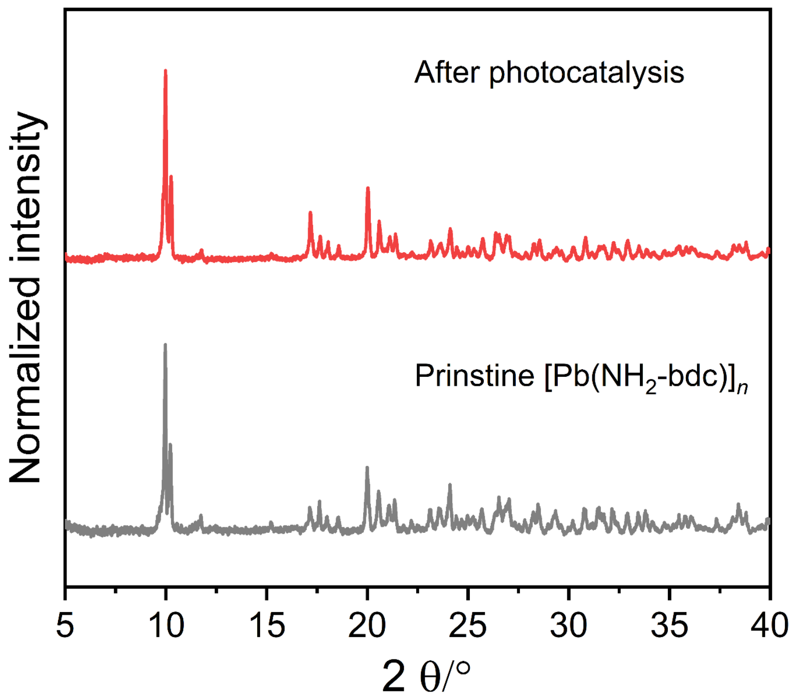


**Supplementary Figure 49 | Photocatalytic stability of [Pb($NH_2$-bdc)]$_n$.** PXRD patterns of [Pb($NH_2$-bdc)]$_n$ before and after photocatalysis.

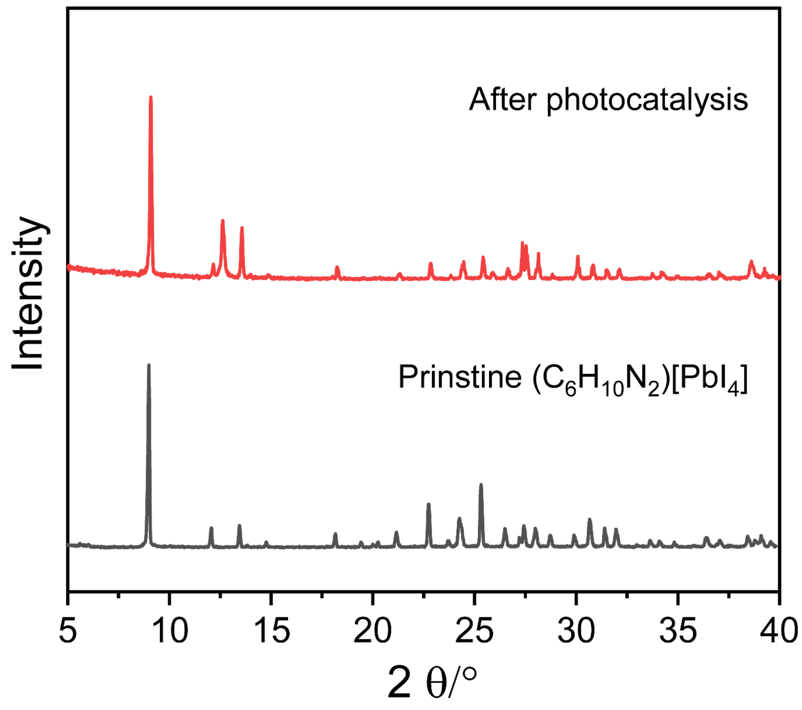


**Supplementary Figure 50 | Photocatalytic stability of ($C_6H_{10}N_2$)[$PbI_4$].** PXRD patterns of ($C_6H_{10}N_2$)[$PbI_4$] before and after photocatalysis.

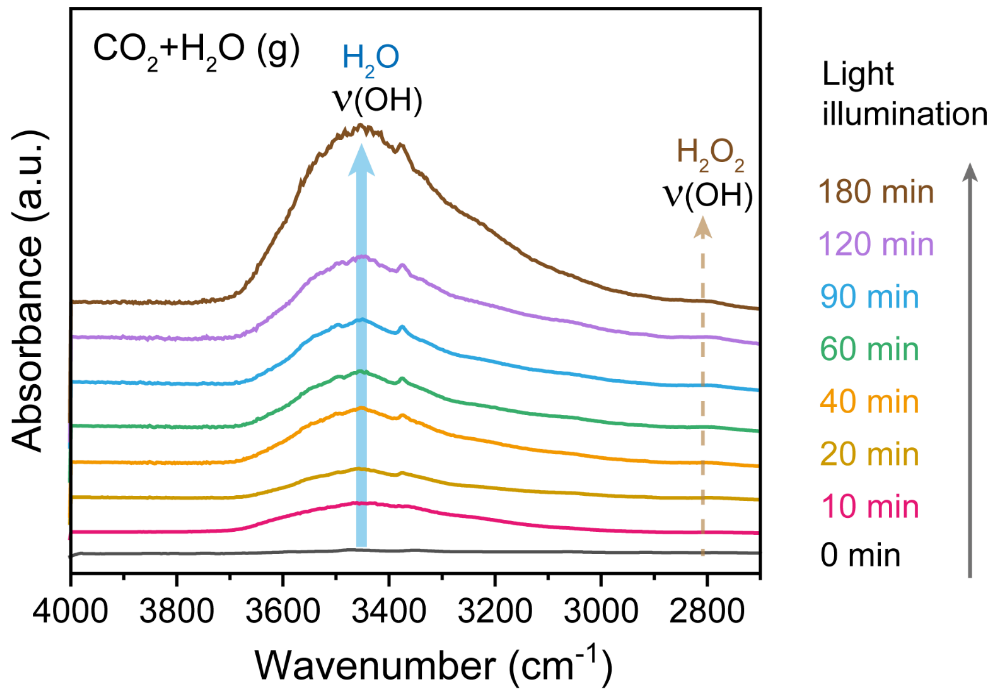


**Supplementary Figure 51 | In situ DRIFTS measurements.** Magnified in-situ DRIFTS spectra for co-adsorption of a mixture of $CO_2$ and $H_2O$ vapor by TMOF-10-$NH_2$(I) in the range of 2700−4000 $cm^{-1}$.

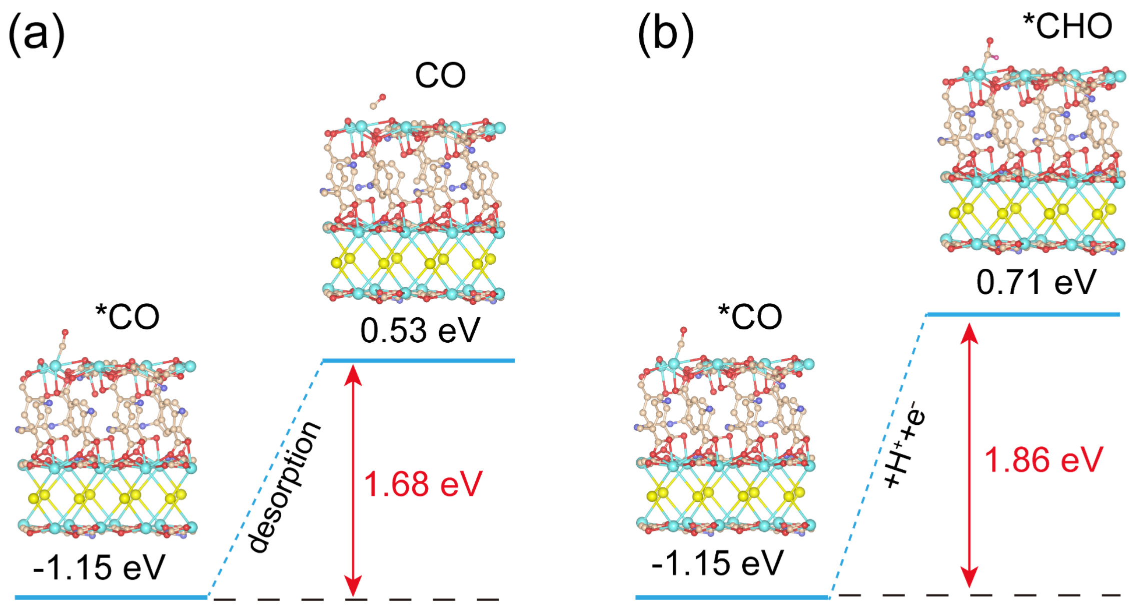


**Supplementary Figure 52 | Free energy diagrams of possible reaction pathways.** Calculated energy barrier for CO desorption (a) and *CHO generation (b) from *CO respectively.

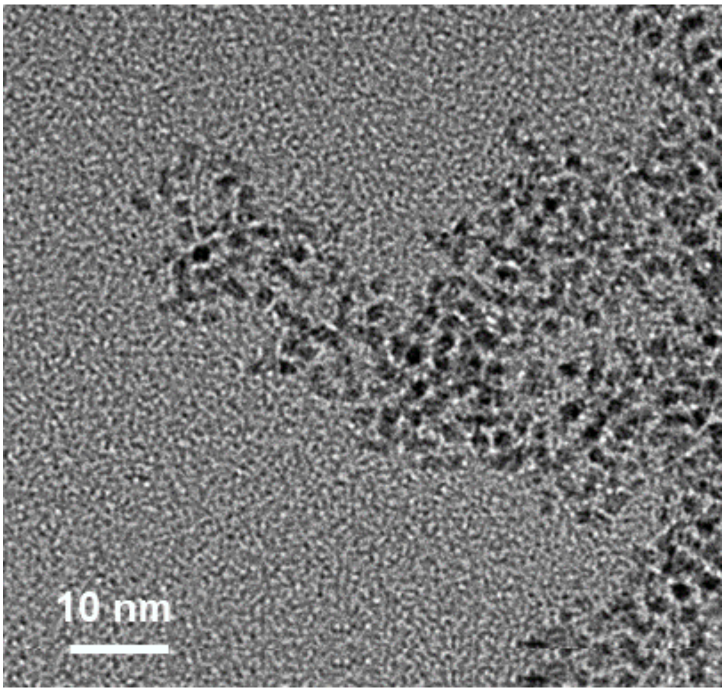


**Supplementary Figure 53 | TEM of Ru NPs.** TEM image of as-synthesized Ru NPs.

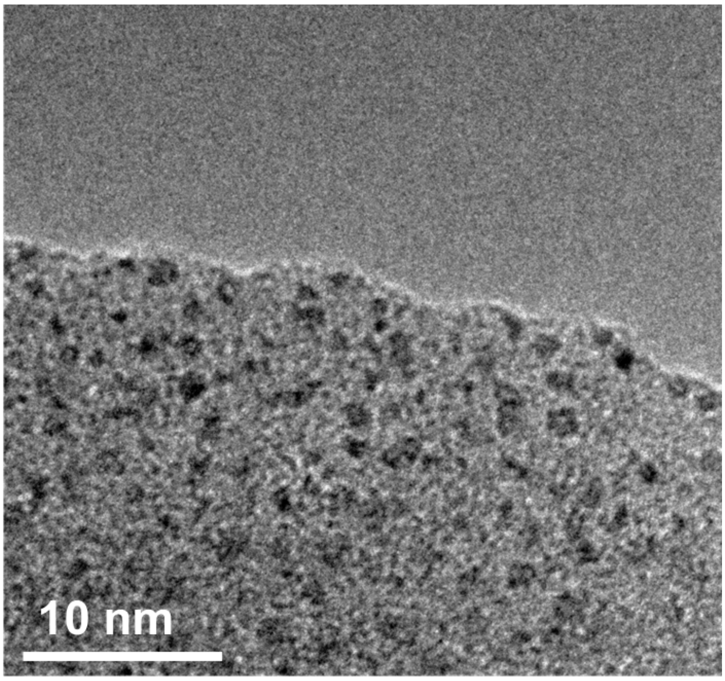


**Supplementary Figure 54 | TEM of Ru@TMOF-10-$NH_2$(I).** TEM image of Ru@TMOF-10-$NH_2$(I) after photocatalysis.

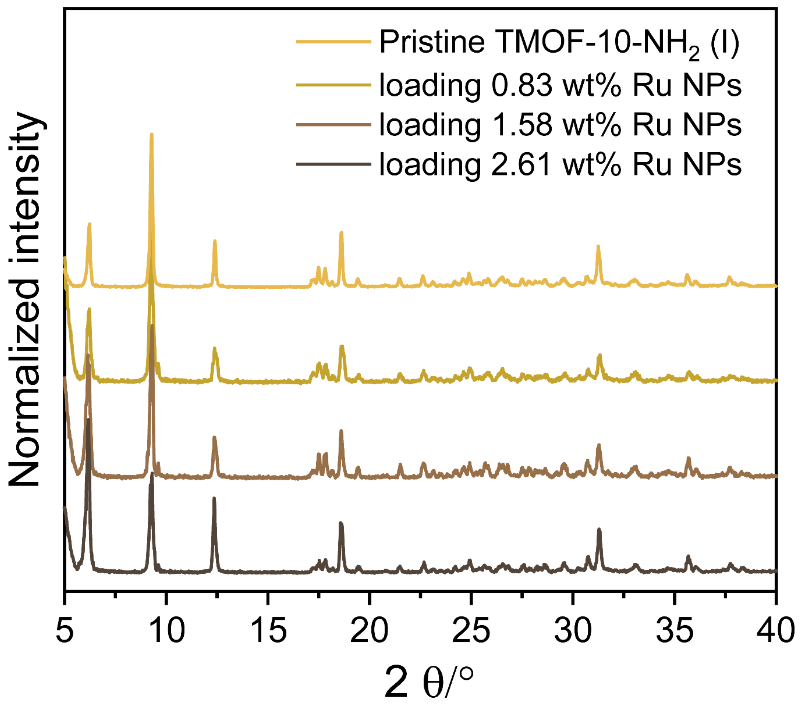


**Supplementary Figure 55 | PXRD patterns of Ru@TMOF-10-$NH_2$(I) and TMOF-10-$NH_2$(I).** Comparison of PXRD patterns of pristine TMOF-10-$NH_2$(I) and at different mass loadings of Ru NPs.

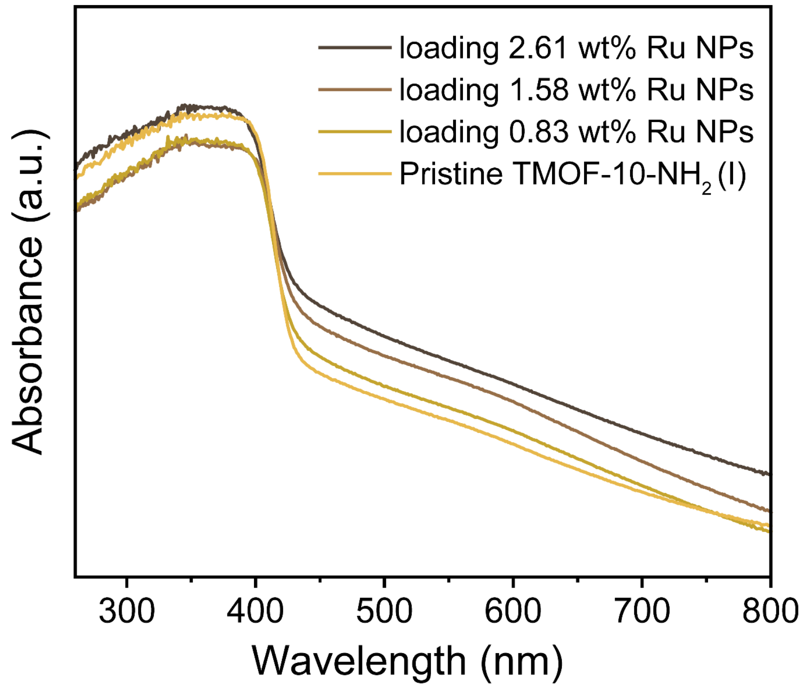


**Supplementary Figure 56 | UV-Vis DRS of Ru@TMOF-10-$NH_2$(I) and TMOF-10-$NH_2$(I).** Comparison of UV-Vis DRS of pristine TMOF-10-$NH_2$(I) and at different mass loadings of Ru NPs.

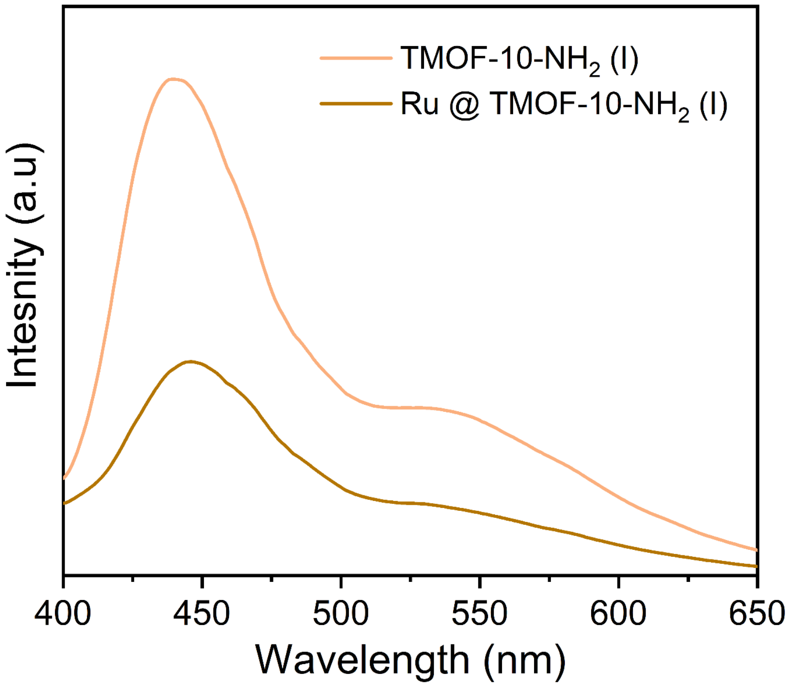


**Supplementary Figure 57 | PL spectra of TMOF-10-NH$_2$(I) and Ru@TMOF-10-NH$_2$(I).** Comparison of PL spectra of TMOF-10-NH$_2$(I) and Ru@TMOF-10-NH$_2$(I) at room temperature.

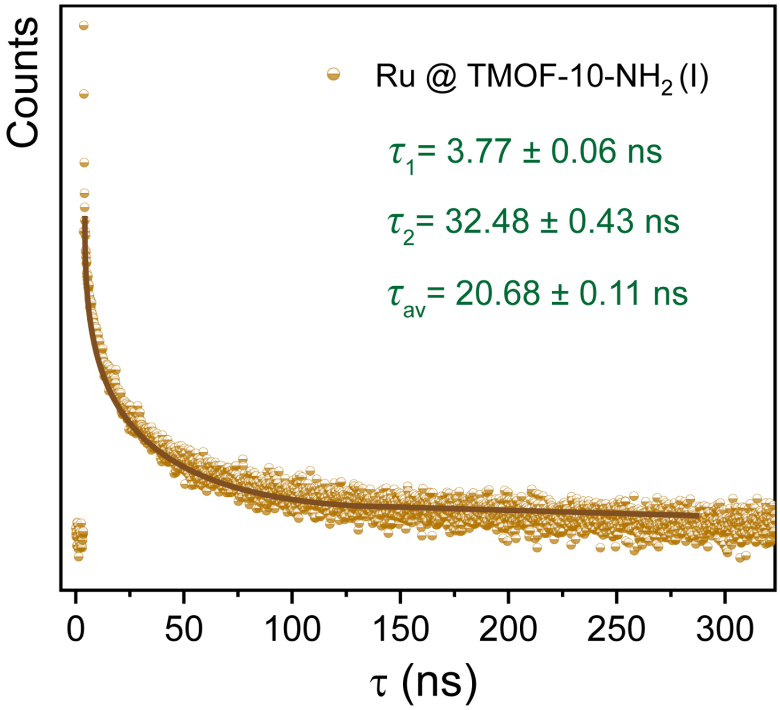


**Supplementary Figure 58 | PL decay curve of Ru@TMOF-10-NH$_2$(I).** PL decay curve of Ru@TMOF-10-NH$_2$(I) with biexponential fit at room temperature.

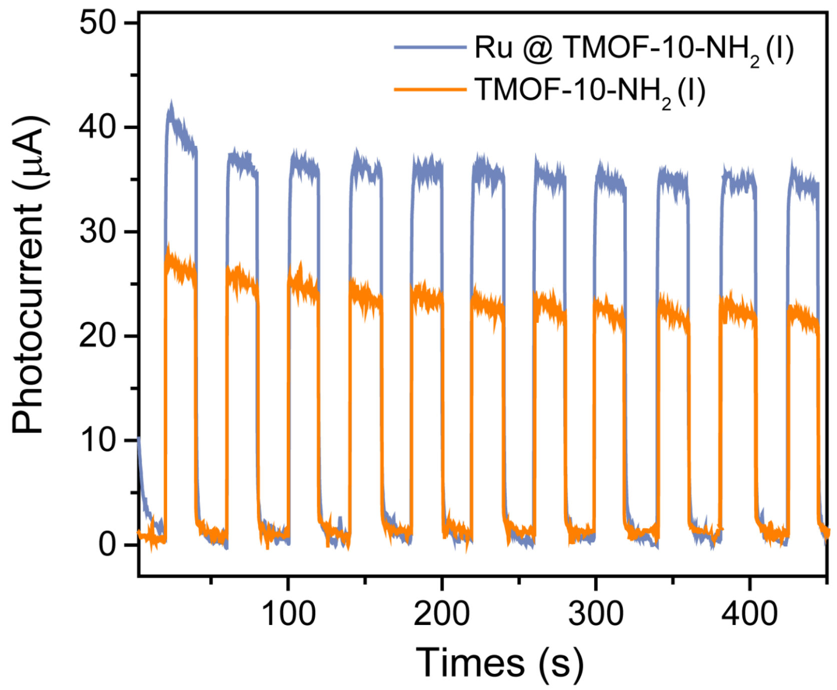


**Supplementary Figure 59 | Photocurrent measurements.** Photocurrent responses of TMOF-10-$NH_2$(I) and Ru@TMOF-10-$NH_2$(I).

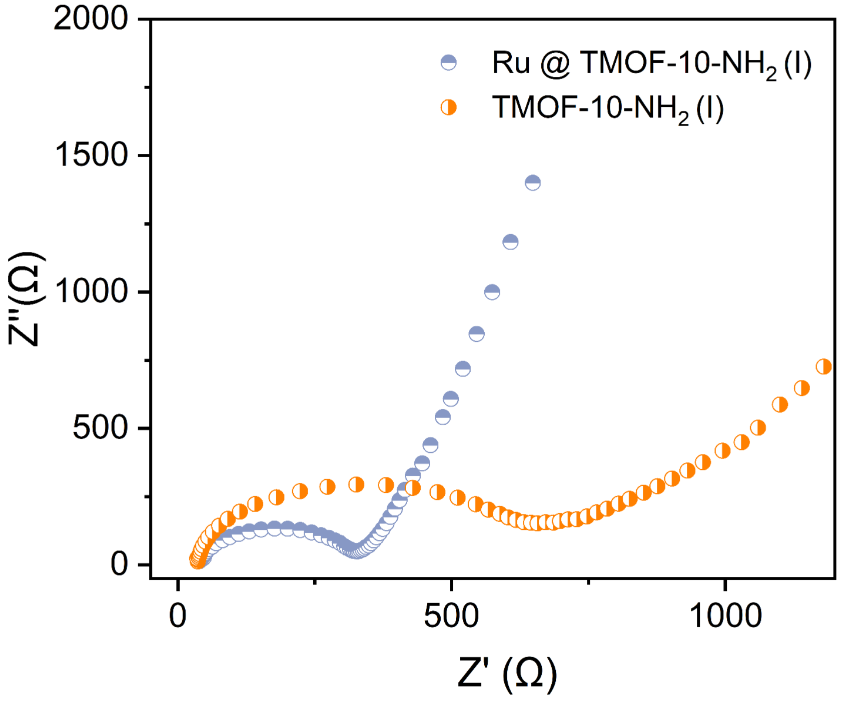


**Supplementary Figure 60 | Electrochemical impedance spectroscopy (EIS) measurements.** EIS Nyquist plots of TMOF-10-$NH_2$(I) and Ru@TMOF-10-$NH_2$(I).

## Supplementary References


1. Yan, S. *et al.* Co-ZIF-9/$TiO_2$ nanostructure for superior $CO_2$ photoreduction activity. *J. Mater. Chem A* **4**, 15126-15133 (2016).

2. Wang, Y. *et al.* In situ self-assembly of zirconium metal–organic frameworks onto ultrathin carbon nitride for enhanced visible light-driven conversion of $CO_2$ to CO. *J. Mater. Chem A* **8**, 6034-6040 (2020).

3. Liu, Q. *et al.* ZIF-8/$Zn_2GeO_4$ nanorods with an enhanced $CO_2$ adsorption property in an aqueous medium for photocatalytic synthesis of liquid fuel. *J. Mater. Chem A* **1**, 11563-11569 (2013).

4. Crake, A., Christoforidis, K. C., Kafizas, A., Zafeiratos, S. & Petit, C. $CO_2$ capture and photocatalytic reduction using bifunctional $TiO_2$/MOF nanocomposites under UV-vis irradiation. *Appl. Catal. B* **210**, 131-140 (2017).

5. Wang, M., Wang, D. & Li, Z. Self-assembly of CPO-27-Mg/$TiO_2$ nanocomposite with enhanced performance for photocatalytic $CO_2$ reduction. *Appl. Catal. B* **183**, 47-52 (2016).

6. Li, R. *et al.* Integration of an inorganic semiconductor with a metal–organic framework: a platform for enhanced gaseous photocatalytic reactions. *Adv. Mater.* **26**, 4783-4788 (2014).

7. Fang, Z.-B. *et al.* Boosting interfacial charge-transfer kinetics for efficient overall $CO_2$ photoreduction via rational design of coordination spheres on metal–organic frameworks. *J. Am. Chem. Soc.* **142**, 12515-12523 (2020).

8. Dong, L. Z. *et al.* Stable heterometallic cluster-based organic framework catalysts for artificial photosynthesis. *Angew. Chem. Int. Ed.* **59**, 2659-2663 (2020).

9. Chen, E.-X. *et al.* Energy band alignment and redox-active sites in metalloporphyrin-spaced metal-catechol frameworks for enhanced $CO_2$ photoreduction. *Angew. Chem. Int. Ed.* **61**, e202111622 (2022).

10. Shyamal, S., Dutta, S. K. & Pradhan, N. Doping iron in $CsPbBr_3$ perovskite nanocrystals for efficient and product selective $CO_2$ reduction. *J. Phys. Chem. Lett.* **10**, 7965-7969 (2019).

11. Zhu, J. *et al.* Synthesis of monodisperse water-stable surface Pb-rich $CsPbCl_3$ nanocrystals for efficient photocatalytic $CO_2$ reduction. *Nanoscale* **12**, 11842-11846 (2020).

12. Mu, Y. F. *et al.* Water‐tolerant lead halide perovskite nanocrystals as efficient photocatalysts for visible‐light‐driven $CO_2$ reduction in pure water. *ChemSusChem* **12**, 4769-4774 (2019).

13. Jiang, Y. *et al*. Hierarchical $CsPbBr_3$ nanocrystal-decorated ZnO nanowire/macroporous graphene hybrids for enhancing charge separation and photocatalytic $CO_2$ reduction. *J. Mater. Chem A* **7**, 13762-13769 (2019).

14. Jiang, Y. *et al*. All-solid-state Z-scheme $\alpha$-$Fe_2O_3$/amine-RGO/$CsPbBr_3$ hybrids for visible-light-driven photocatalytic $CO_2$ reduction. *Chem* **6**, 766-780 (2020).

15. Xu, Y.-F. *et al*. Enhanced solar-driven gaseous $CO_2$ conversion by $CsPbBr_3$ nanocrystal/Pd nanosheet Schottky-junction photocatalyst. *ACS Appl. Energy Mater.* **1**, 5083-5089 (2018).

16. Wu, L. Y. *et al*. Encapsulating perovskite quantum dots in iron-based metal–organic frameworks (MOFs) for efficient photocatalytic $CO_2$ reduction. *Angew. Chem. Int. Ed.* **58**, 9491-9495 (2019).

17. Kong, Z.-C. *et al*. Core@shell $CsPbBr_3$@zeolitic imidazolate framework nanocomposite for efficient photocatalytic $CO_2$ reduction. *ACS Energy Lett.* **3**, 2656-2662 (2018).
